# Development of a 2,4-Diaminothiazole Series for the Treatment of Human African Trypanosomiasis Highlights the Importance of Static–Cidal Screening of Analogues

**DOI:** 10.1021/acs.jmedchem.3c00509

**Published:** 2023-06-21

**Authors:** Laura A. T. Cleghorn, Richard J. Wall, Sébastien Albrecht, Stuart A. MacGowan, Suzanne Norval, Manu De Rycker, Andrew Woodland, Daniel Spinks, Stephen Thompson, Stephen Patterson, Victoriano Corpas Lopez, Gourav Dey, Iain T. Collie, Irene Hallyburton, Robert Kime, Frederick R. C. Simeons, Laste Stojanovski, Julie A. Frearson, Paul G. Wyatt, Kevin D. Read, Ian H. Gilbert, Susan Wyllie

**Affiliations:** † Drug Discovery Unit, Wellcome Centre for Anti-infectives Research, University of Dundee, Dow Street, Dundee DD1 5EH, U.K.; ‡ Wellcome Centre for Anti-infectives Research, School of Life Sciences, University of Dundee, Dow Street, Dundee DD1 5EH, U.K.; § Division of Computational Biology, School of Life Sciences, University of Dundee, Dow Street, Dundee DD1 5EH, U.K.

## Abstract

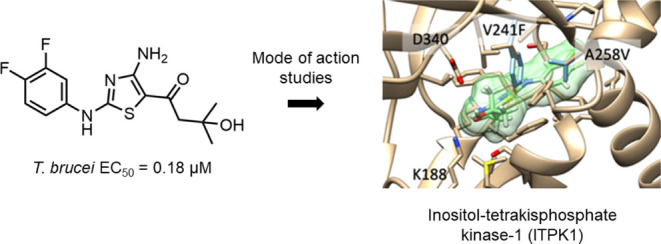

While treatment options for human African trypanosomiasis (HAT) have improved significantly, there is still a need for new drugs with eradication now a realistic possibility. Here, we report the development of 2,4-diaminothiazoles that demonstrate significant potency against *Trypanosoma brucei*, the causative agent of HAT. Using phenotypic screening to guide structure–activity relationships, potent drug-like inhibitors were developed. Proof of concept was established in an animal model of the hemolymphatic stage of HAT. To treat the meningoencephalitic stage of infection, compounds were optimized for pharmacokinetic properties, including blood–brain barrier penetration. However, in vivo efficacy was not achieved, in part due to compounds evolving from a cytocidal to a cytostatic mechanism of action. Subsequent studies identified a nonessential kinase involved in the inositol biosynthesis pathway as the molecular target of these cytostatic compounds. These studies highlight the need for cytocidal drugs for the treatment of HAT and the importance of static–cidal screening of analogues.

## Introduction

Human African trypanosomiasis (HAT), also known as African sleeping sickness, is a parasitic disease caused by infection with *Trypanosoma brucei gambiense* and *T. b. rhodesiense*. Around 70 million people in 36 sub-Saharan African countries are at risk of contracting HAT. This neglected disease is transmitted by the bite of a tsetse fly and can be fatal if not treated. Cases and deaths attributed to HAT have gradually decreased over the past few decades, with only 663 infections reported in 2020.^1^ This precipitous drop in cases has raised hopes that eradication of HAT could be within reach; however, a similar reduction in cases was achieved in the early 1960s before again surging due to failures in surveillance and a lack of treatment options.^2,3^

There are two stages of HAT; in stage 1, trypanosomes rapidly multiply in host subcutaneous tissues, blood, and the lymphatic system, resulting in bouts of fever, headache, joint pain, and itching. In the second stage of the disease, parasites cross the blood–brain barrier to infect the central nervous system, causing confusion, sensory disturbance, poor coordination, and disruption of the sleep cycle. This meningoencephalitic stage of infection is fatal if left untreated.^4^ Affected populations commonly live in remote areas with limited access to adequate health care, impeding rapid diagnosis and treatment. Thus, a minimum requirement for any new HAT treatment would be the ability to treat both stages of the disease.^5^

Treatment regimens for HAT vary depending on the stage of the disease at diagnosis and the species of the parasite responsible for the infection. More than 98% of cases are caused by infection with *T. b. gambiense*. The standard of care for stage 1 and 2 *gambiense* infection is either the newly registered oral drug fexindazole,^6^ pentamidine, or nifurtimox–eflornithine combination treatment depending on the age, weight, and white blood cell count of the patient.^1^ For chronic stage *T. b. rhodesiense* infections, the frontline therapy is either intravenous suramin or pentamidine, while the only treatment available for stage 2 remains the highly toxic arsenical melarsoprol. Clearly, improved treatment options for *T. b. rhodesiense* infections would be highly desirable.

Previously, we reported the results of a high-throughput screening (HTS) campaign to identify inhibitors of *T. brucei* glycogen synthase kinase 3 (*Tb*GSK3) with the aim of chemically validating this kinase as a viable drug target in the African trypanosome.^7^ In the course of this study, a ∼4100 compound library of kinase inhibitor scaffolds was screened against *Tb*GSK3.^8^ From this screen, a diaminothiazole series of compounds were developed, demonstrating nanomolar activity against both *Tb*GSK3 and bloodstream trypanosomes. As this series evolved, it became apparent that the potency of later compounds was no longer principally driven through inhibition of *Tb*GSK3, suggesting that an alternative mechanism of action may be involved. This divergent series was subsequently optimized using phenotypic activity against bloodstream trypanosomes as the principal driver, with a counter screen against mammalian cells (MRC-5) used to monitor selectivity.

Here, we report the evolution of this diaminothiazole series into low nanomolar inhibitors of *T. brucei*, with the lead compound (**38**) capable of penetrating the blood–brain barrier and demonstrating efficacy in a model of stage 1 infection. Unfortunately, this activity did not translate into models of meningoencephalitic infection. Comprehensive mechanism of action and drug target deconvolution studies confirm that compound **38** no longer inhibits *Tb*GSK3 but rather targets a kinase (inositol-tetrakisphosphate 1-kinase, putative) involved in the inositol biosynthetic pathway. Inositol-tetrakisphosphate 1-kinase is not essential for parasite survival; thus, treatment with compound **38** is cytostatic rather than cytocidal, explaining the failure of this diaminothiazole to cure animal models of infection. The challenges faced and lessons learned from this study using phenotypic activity to drive the development of structure–activity relationship (SAR) in this series are discussed.

## Results and Discussion

### Starting Point

**Figure 1:**
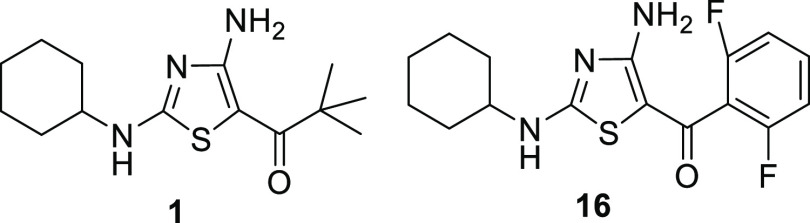
Chemical evolution of compounds **1** to **16**.

Compound **1** was selected as the starting point for this program focused on using phenotypic activity against bloodstream-form (BSF) *T. brucei* as the principal driver for development. As previously established,^7^ this compound demonstrated modest activity against *Tb*GSK3 in assays with the recombinant enzyme (IC_50_ = 12 μM); however, it was a potent inhibitor of the growth of *T. brucei* in vitro (EC_50_ = 260 nM). This 70-fold shift in potency between cell-based and enzymatic assays strongly suggested that potency was no longer due to inhibition of GSK3 and that compound **1** was likely interacting with an alternative molecular target(s). Compound **1** retained excellent selectivity (∼200-fold) over the mammalian MRC-5 cell line counter screen. In addition, in silico models (StarDrop) predicted that compound **1** would penetrate the blood–brain barrier (PSA = 68 Å^2^; MW = 281) and thus may have utility in treating stage 2 HAT. Early structure–activity work established that the *t*-butyl group could be replaced with 2,6-difluorophenyl that displayed promising in vitro potency (*T. brucei* EC_50_ = 0.11 μM) (Figure 1).

### Pharmacokinetic Studies—Compound **16** and Stage 1 Efficacy Studies

**Figure 2:**
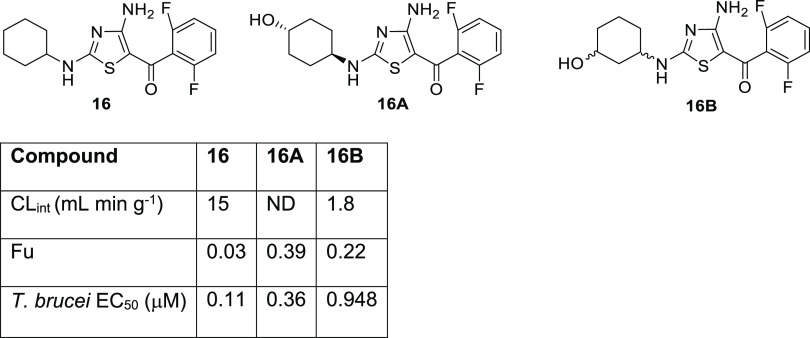
Putative metabolites of compound **16**.

Based on an initial low intrinsic clearance when **16** was incubated with mouse liver microsomes, the pharmacokinetic properties of **16** were profiled with a view to progressing this compound to a proof-of-concept study in a rodent model of disease. In female NMRI mice dosed via oral (PO) and intraperitoneal (IP) administration, the exposure of compound **16** was found to be relatively poor (Table 1), most likely due to first-pass P450 metabolism. To try to increase exposure in order to achieve in vivo proof of concept in a mouse model of stage 1 efficacy, we reassessed compound **16** exposure in HRN mice^9−11^ following IP administration. These transgenic mice are hepatic cytochrome P450 reductase null and consequently lack P450-driven metabolism in the liver. In IP-dosed HRN mice, exposure to compound **16** was considerably higher (15-fold) than that achieved when dosing NMRI mice via the same route (Table 1). These data confirm that clearance by CYP450 metabolism in the liver is largely responsible for the poor exposure observed for compound **16** in NMRI mice. This was further supported by the high mouse microsomal intrinsic clearance upon repeat incubation (Figure 2).

**Table 1:** Pharmacokinetic Studies on **16**

species	route	dose (mg kg^–1^)	*C*_max_ (ng mL^–1^)^a^	*T*_max_ (h)^b^	AUC (ng mL h^–1^)^c^
NMRI mice	PO	10	33	0.50	5700
NMRI mice	IP	10	830	0.25	66,000
HRN mice	IP	10	3800	0.50	1,000,000

a *C*_max_ is the maximum concentration reached.

b *T*_max_ is the time after the initial dose at which the maximum concentration was reached.

c AUC is the area under the curve.

Compound **16** progressed to a stage 1 efficacy study in HRN mice, with IP dosing at 10 mg kg^–1^ twice daily for 4 days. The NMRI mouse efficacy study using the same dose route and regimen was also carried out to demonstrate the utility of the HRN mouse for rapid proof of concept. Cure in these models of infection was defined as no signs of parasitemia for 30 days following dosing. Surprisingly, in the NMRI model, dosing with compound **16** effected complete cure with no sign of relapse throughout the 30 day study. In contrast, in HRN mice where compound **16** reached considerably higher exposure, compound-dependent toxicity was observed, with only one out of three animals cured.

Due to poor exposure in NMRI mice following IP administration at 10 mg kg^–1^, free blood concentrations did not reach the EC_50_ or EC_90_. Total blood concentrations were only above the EC_50_ and EC_90_ for 3 and 2.5 h, respectively. However, this compound was efficacious in this stage 1 model of HAT. This is counter to the usual experience for a nonreactive small molecule treating parasitic diseases where time above free drug EC_90_ or higher in blood and brain is usually required to drive efficacy unless compound accumulation is occurring in the parasite through, for example, active uptake. Consequently, and because of the unexpected stage 1 efficacy observed in NMRI mice, compound **16** also progressed to a stage 2 model of CNS disease. Compound **16** had good brain penetration (B/B ratio 3.3) but a very low brain free fraction (Fu = 0.0063) such that the brain free concentration did not reach EC_50._ Thus, it was perhaps less surprising that the compound was not efficacious in the stage 2 disease model.

The observed stage 1 efficacy of compound **16** could be explained if the molecular target(s) of compound **16** required only transient inhibition to cause cell death, potentially by triggering some form of a lethal cascade. It is also possible that partial inhibition of an enzyme catalyzing the rate-determining step of a pathway may also result in cell death. However, the lack of efficacy in the stage 2 model perhaps argues against this hypothesis. Alternatively, the cures observed in NMRI-treated mice may be due to an active metabolite, formed by P450 metabolism, which is not formed in P450 reductase-deficient HRN mice and has sufficient free concentrations to deliver efficacy. Studies to investigate these two scenarios are described below. Regardless, our studies provided proof of concept that these diaminothiazoles can deliver in vivo efficacy in a stage 1 model of disease. Addressing the poor oral exposure and narrow therapeutic window of compound **16** (dosing at 20 mg kg^–1^ was not tolerated) became our focus for medicinal chemistry optimization.

### Metabolite Identification Studies with Compound **16**

To address the possibility that the efficacy of **16** in the NMRI mouse model was due to the generation of an active metabolite, preliminary metabolite identification studies were carried out. Compound **16** (0.5 and 5 μM) was incubated with mouse liver microsomes for 60 min. Following incubation, two metabolite peaks were observed with retention times of 3.43 and 3.86 min following LC–MS analysis; both with a *m*/*z* of 354 (MW **16** = 337), indicating that compound **16** had become oxidized [addition of OH (+17 Da)]. A P450 metabolism model (StarDrop, cytochrome P450 metabolizing isoforms 2C9, 2D6, and 3A4) was used to predict the regions of **16** most susceptible to metabolism. The predicted metabolites were synthesized, and their retention times were compared to those of the metabolites observed after incubation of **16**. The 3.43 min peak corresponded to 4-hydroxycyclohexyl (**16A**), and 3-hydroxycyclohexyl (**16B**) corresponded to the 3.86 min peak. Next, the potency of these metabolites was determined against *T. brucei*, and PK properties were profiled. While neither metabolite demonstrated improved potency compared to the parent compound (Figure 2), the free fraction of both metabolites increased with the addition of hydroxy groups. The in vivo concentration–time profile of the metabolites in NMRI mice was not assessed, so the possibility that the efficacy of **16** was due to an active metabolite(s) remains only a hypothesis.

### Lead Optimization

The data from our efficacy studies with compound **16** provided sufficient confidence to initiate a focused medicinal chemistry program. The aim of this study was to increase potency, increase microsomal stability (ideally a CL_int_ < 1 mL min g^–1^), and improve selectivity. To address the metabolic issues observed with **16**, we worked on reducing the log *P* and adding blocking groups at positions perceived as metabolically labile. Initial hit expansion efforts explored a range of substituents in the R^1^ and R^2^ positions to examine the effect on the antiproliferative activity observed and to test the limits of the unknown binding pocket (see Table 2 for the positions of R^1^ and R^2^ substituents).

**Table 2:**
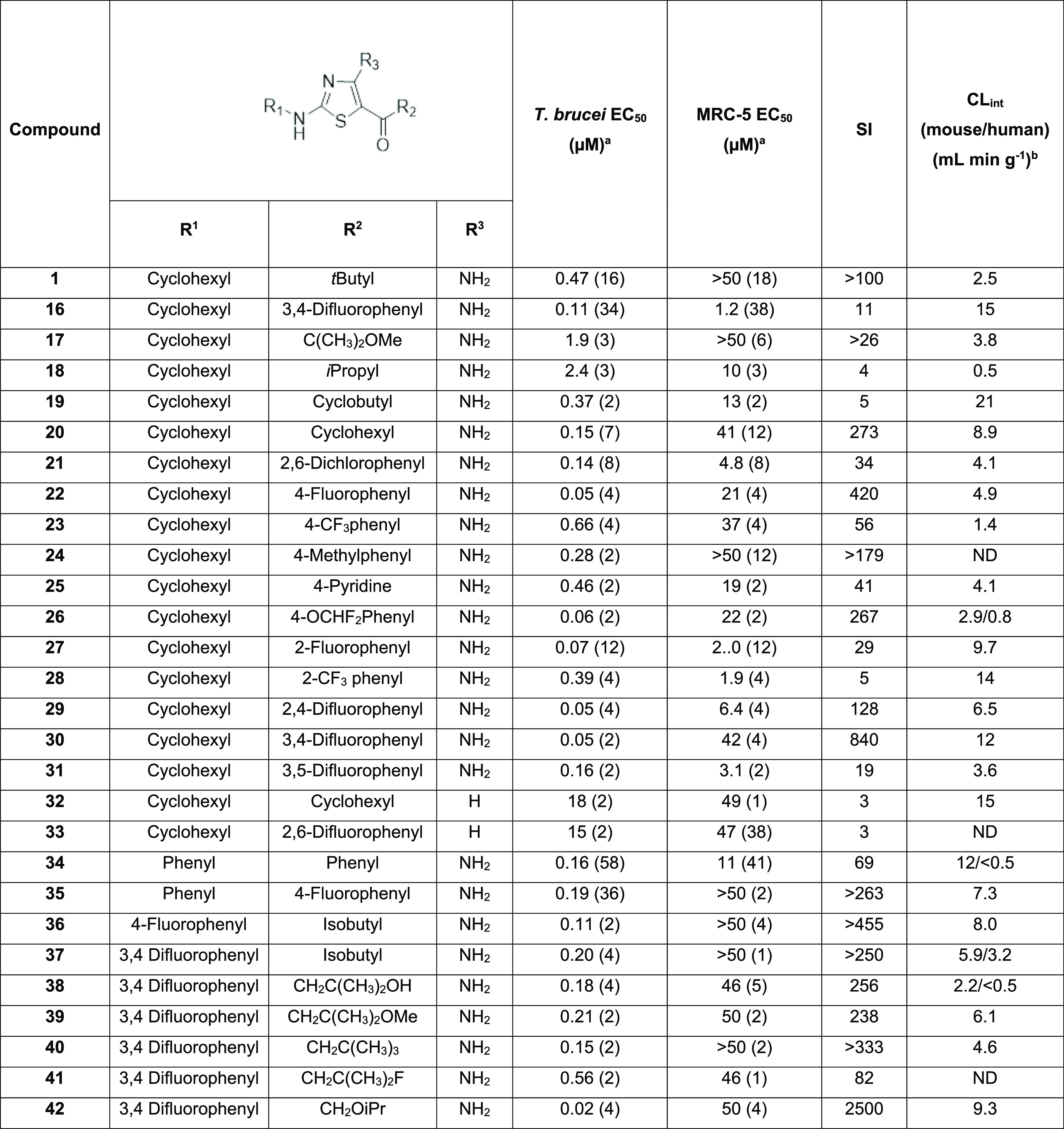
Initial Hit Expansion

a EC_50_ values are shown as mean values of two or more determinations.

b CL_int_ is the mouse liver microsomal intrinsic clearance. The standard deviation is typically within 2–3-fold of the EC_50_. ND—not determined. SI—selectivity index (EC_50_ MRC5/EC_50_*T. brucei*).

The target product profile (TPP) for HAT stipulates that any new drug should be effective against the meningoencephalitic stage of infection and thus must be capable of crossing the blood–brain barrier. CNS penetration is driven by a complex interplay of physicochemical properties, including molecular weight, polar surface area (PSA), and lipophilicity (clog *P*). This work was initiated before the widespread use of multiparameter optimization scores.^12^ However, the focus was on retaining low molecular weight and PSA combined with a clog *P* of around 3. Physicochemical properties are documented in Table S1.

We investigated several scenarios to optimize the compounds: (i) R^1^ and R^2^ aliphatic, (ii) R^1^ aliphatic and R^2^ aromatic, and (iii) R^1^ aromatic and R^2^ aliphatic.

(i) R^1^ and R^2^ aliphatic: to test the scope of the R^2^ position, initial work focused on analogues retaining R^1^ as cyclohexyl and varying R^2^ (Table 2, compounds **1** and **17–33**). Replacement of one of the methyl groups of the ^*t*^Bu on **1** with methoxy **17** and removal of a methyl group **18** was detrimental to potency against *T. brucei* and in the case of the *iso*-propyl, exacerbated toxicity in the mammalian cell counter screen. Cyclobutyl **19** was equipotent with compound **1** but less selective in the mammalian counter screen combined with a large increase in microsomal turnover (**1** CL_int_ = 2.5 mL min g^–1^, **19** CL_int_ = 21 mL min g^–1^). Cyclohexyl at R^2^ resulted in a 3-fold increase in potency with a similar level of selectivity. A reasonable picture of SAR had been built up through maintaining R^1^ as cyclohexyl. Although modest improvements in potency were achieved, there was still a need for improved microsomal stability to provide a suitable candidate for further study.
(ii) R^1^ aliphatic and R^2^ aromatic: swapping R^2^ from aliphatic to aromatic retained potency but reduced selectivity in the mammalian counter screen (2,6-dichloro **21** and 2,6-difluoro **16**). In general, this could be mitigated by substitution at the 4-position of the aromatic ring of R^2^ (**22**, **23**, **24**, **25**, and **26**). Our hypothesis was the observed toxicity was due to inhibition of a mammalian cyclin-dependent kinase as structures with a similar pharmacophore have been reported in the literature,^13,14^ with the phenyl ring presumably sitting inside an ATP binding pocket. The 3,4-difluoro substitution is well tolerated (**30)**; however, the symmetrical 3,5-disubstitution (**31)** without the presence of a blocking group at the 4-position shows reasonable potency but lower selectivity. The reduction in toxicity with a substituent in the 4-position was probably due to either a steric clash between the 4-substituent and the protein or the blocking interaction between the aromatic C–H and the mammalian protein. Good potency was observed with monosubstitution in the 2-position of the R^2^ aromatic ring (2-fluoro **27** and 2-trifluoromethyl **28**), although selectivity compared to MRC-5 cells was relatively poor. Addition of a fluoro in the 4-position, to give the 2,4-difluoro-analogue (**29** and **30**), retained potency and slightly improved selectivity, although not to the level of the 4-fluoro derivative (**22**). Removing the free amino group at the 4-position on the thiazole was not tolerated with a >20-fold reduction in potency (**32** and **33**), indicating that diamino substitution may be important for key donor–acceptor–donor interactions in the final binding pose that is adopted.
(iii) R^1^ aromatic and R^2^ aliphatic: we then explored replacing the cyclohexyl at R^1^ with a phenyl ring. Compound **34** with R^1^ and R^2^ as phenyl demonstrated reasonable potency alongside a selectivity window of ∼100-fold. Similarly, good potency and selectivity were observed when R^1^ was phenyl and R^2^ was 4-fluorophenyl (**35**). In an attempt to improve DMPK properties such as microsomal turnover and solubility, we next investigated the impact of aromatic and aliphatic substitutions at positions R^1^ and R^2^, respectively. The initial compound (**37**), with an isobutyl substituent at R^2^, showed good potency and selectivity but was rapidly turned over in microsomes. The addition of a free alcohol (**38**) to the isobutyl group of R^2^ resulted in a compound that was equipotent with **37** but less susceptible to microsomal turnover. Compound **38** could be capped with a methyl group (**39**) without affecting potency, although perhaps unsurprisingly, the microsomal turnover was increased. A similar result was obtained by replacing the methoxy (**40**) with a methyl. Swapping the hydroxyl for a fluoro (**41**) led to a slight reduction in potency but offered no advantage in terms of physiochemical parameters over **38**. A considerable jump in potency was observed by the introduction of an oxygen atom to form an ether moiety; the isobutoxy derivative (**42**) showed a 10-fold improvement in activity, although the microsomal turnover was still high.

### Lead Development

Further work focused on exploring the SAR around the ether moiety at R^3^ while maintaining R^1^ as aromatic (predominantly 3,4-difluorophenyl; Table 3). Our aim was to reduce the microsomal turnover.

**Table 3:**
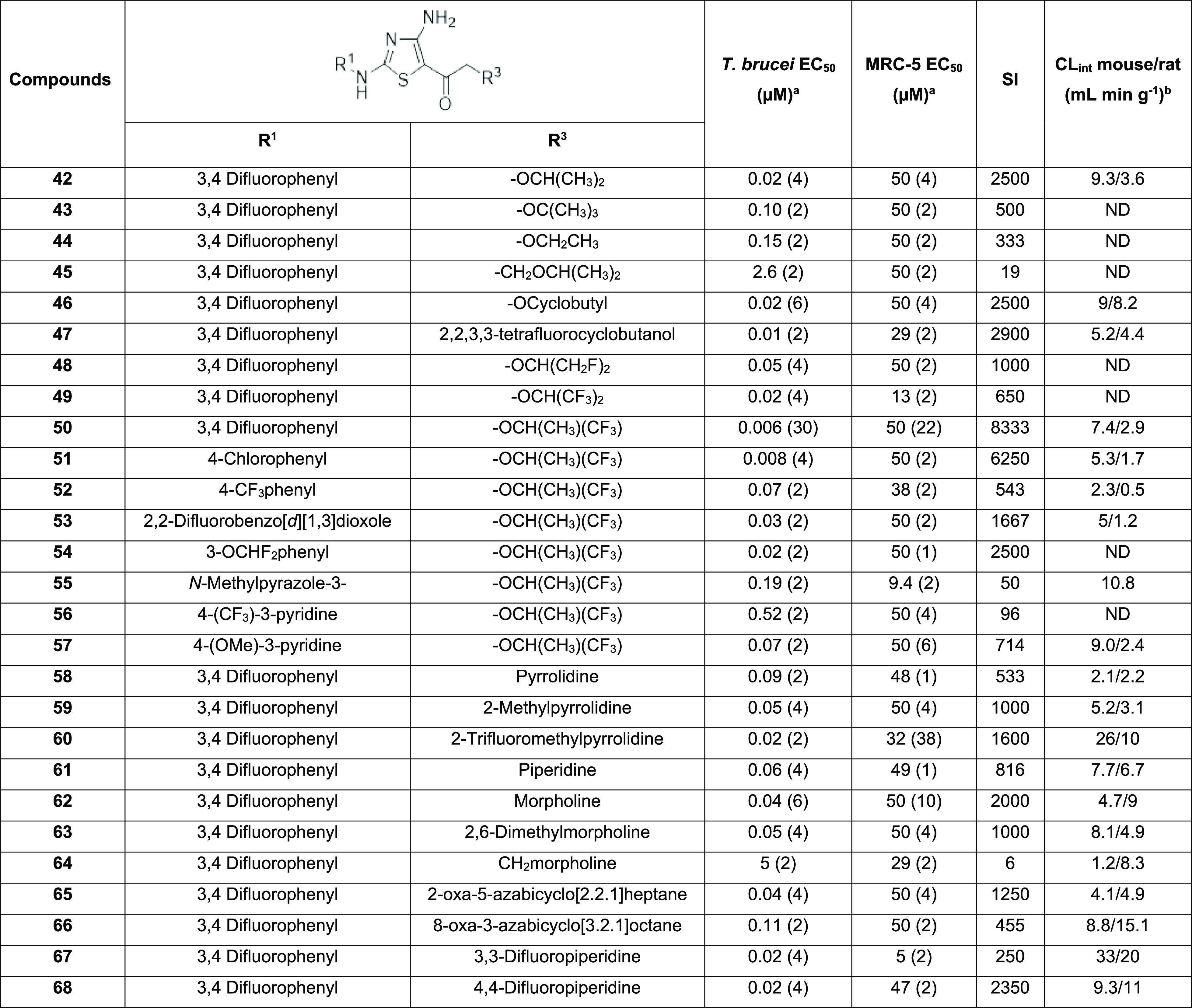
Lead Development

a EC_50_ values are shown as mean values with the number of experimental replicates shown in parentheses.

b CL_int_ is the mouse liver microsomal intrinsic clearance. SI—selectivity index (EC_50_ MRC5/EC_50_*T. brucei*). Standard deviation is typically within 2–3-fold of the EC_50_. ND—not determined.

Changes to the steric bulk of R^3^ were generally detrimental to activity. Thus, increasing size (^*t*^butyloxy **43**, EC_50_ = 0.10 μM), decreasing size (ethyloxy, **44**, EC_50_ = 0.15 μM), or extending chain length (**45**, EC_50_ = 2.6 μM) all showed a drop in potency compared to the isopropyl derivative (**42**, EC_50_ = 0.02 μM). The cyclobutyl (**46**) and 2,2,3,3-tetrafluorocyclobutyl (**47**) derivatives were equipotent, with the latter demonstrating a slight increase in microsomal stability but also a slight increase in toxicity in the mammalian counter screen. Fluorinated analogues of **42** either monofluoro on each methyl (**48**) or replacing each methyl with trifluoromethyl (**49**) were well tolerated. Single-digit nM potency (EC_50_ = 6 nM) was achieved against *T. brucei* when one of the methyl groups of the isopropyl was replaced with trifluoromethyl (**50**).

#### Trifluoroethyl Analogues

A range of thiazole analogues with 2-((1,1,1-trifluoropropan-2-yl)oxy)ethanone in the 5-position were investigated and showed good potency and selectivity. 4-Chlorophenyl (**51**) at R^1^ was found to be equipotent with **50**, 4-trifluoromethyl (**52**) improved the microsomal turnover with a 10-fold loss in potency, and 2,2-difluorobenzo[*d*][1,3]dioxole (**53**) and 3-difluoromethoxyphenyl (**54**) were well tolerated but offered no benefit over **50**. A range of heterocyclics were examined in the R^1^ position to look at increasing potential binding interactions in the active site and reducing microsomal turnover while retaining solubility. 4-Methoxy-3-pyridyl (**57**) was well tolerated; however, microsomal turnover was not improved; 4-trifluoromethyl-3-pyridyl (**56**) and the pyrazole (**55**) all showed reduced potency against bloodstream trypanosomes but good levels of solubility >100 μg mL^–1^.

#### Amino Analogues

Retaining 3,4-difluorophenyl at position R^1^, a series of amines were examined as an alternative R^3^ moiety. The addition of an amine in this position generally led to a reduction in clog *P* (Table S1). Pyrrolidine at R^3^ resulted in promising potency, good selectivity, and improved microsomal stability. Substitution of the pyrrolidine (2-methyl, **59**) and (2-trifluoromethyl, **60**) improved potency but at the detriment of microsomal turnover. Piperidine (**61**), morpholine (**62**), and 2,6-dimethylmorpholine (**63**) substitutions were shown to be equipotent with the pyrrolidine analogue, but levels of microsomal turnover remained high. The insertion of a methylene linker between the heterocycle and the cyclic amine was not well tolerated with a >110-fold drop off in potency (**64**). The addition of a bridge to the cyclic ring 2-oxa-5-azabicyclo[2.2.1]heptane (**65**) and 8-oxa-3-azabicyclo[3.2.1]octane (**66**) was well tolerated. 2,2 (**67**) and 3,3-difluoropiperidine (**68**) showed very good potency and selectivity; however, the addition of fluorines onto the aliphatic chain did not improve microsomal turnover.

### Drug Metabolism and Pharmacokinetics

#### Brain/Blood Compound Ratios

Medicines for HAT are required to treat both acute and chronic infections, where parasites pass through the blood/brain barrier and enter the CNS. With this in mind, mice were dosed with an IP bolus of our test compounds, and compound concentrations in blood and brain were determined by UPLC–MS/MS. As the series evolved, brain penetration of the total compound improved considerably with compounds **16** and **38** confirmed to have brain/blood (B/B) ratios of 3.3 and 1.4, respectively. The brain fraction unbound was 0.0063 for **16** and 0.0313 for **38**. Brain penetration of the series as a whole tracked with the generally accepted rules of CNS penetration, with a lower topological PSA (TPSA) and molecular weight compounds having an increased B/B ratio (Table S2 and Figure S1). However, the link between log *P* and the B/B ratio was slightly less convincing. Compounds with the highest B/B ratio had log *D* values of between 3 and 5, with only four compounds from the series falling outside of this range. One compound (**18**, Figure S1) met the above criteria but had a particularly poor ratio of 0.06, while a structurally similar compound, with comparable physicochemical properties (**37**), had an improved ratio of 4.7. This difference could be explained by **18** being a substrate for the P-glycoprotein efflux pump.

In reviewing the entirety of the data generated from our lead development studies, compound **38** was selected for subsequent in vivo assessment. The selection of compound **38** was made based on promising potency against *T. brucei* in vitro, acceptable selectivity over mammalian cells, and good mouse and human microsomal stability. None of the other analogues tested demonstrated sufficient metabolic stability in the pharmacodynamic mouse model (CL_int_ < 5 mL min g^–1^) to allow a meaningful experiment while at the same time providing the required potency and selectivity. Compound **26** had a low free fraction and compound **52** offered no significant advantage over **38** in terms of potency, selectivity, or microsomal stability.

### Pharmacokinetic and Efficacy Studies

Further profiling of compound **38** confirmed that it displayed good aqueous solubility (>100 μg mL^–1^), as well as improved clearance compared to **16** (CL_int_ = 2.2 mL min g^–1^ in mouse). PK/PD parameters for compound **38** were also established in rats. Good levels of oral exposure were achieved in rats, with a later *T*_max_ and slower elimination than in mice (Table 4). Based on our PK data, dosing mice with compound **38** at 50 mg kg^–1^ IP gave a total blood *C*_max_ of 18,000 ng mL^–1^ (55 μM) and a free *C*_max_ of 2600 ng mL^–1^ (7.9 μM). Given the EC_90_ is 131 ng mL^–1^ (0.4 μM), the free peripheral concentration was above EC_90_ for approximately 180 min following a single 50 mg kg^–1^ IP dose. Following a maximum tolerated dose study in NMRI mice, a maximum dose of 100 mg kg^–1^ IP was chosen for this compound. Assuming linearity of exposure, this was extrapolated to a total *C*_max_ of 36,000 ng mL^–1^ (110 μM) and a free *C*_max_ of 5200 ng mL^–1^ (16 μM) and the length of time unbound levels was above EC_90_ was approximately 250 min.

**Table 4:** PK Studies of **38**

species	route	dose (mg kg^–1^)	*C*_max_ (ng mL^–1^)^a^	*T*_max_ (h)^b^	AUC (ng mL h^–1^)^c^
rat	PO	10	1400	4	470,000
NMRI (mice)	IP	50	18,000	0.5	1,900,000

a *C*_max_ is the maximum concentration reached.

b *T*_max_ is the time after the initial dose at which the maximum concentration was reached.

c AUC is the area under the curve.

In male Sprague-Dawley (SD) rats dosed at 10 mg kg^–1^ PO, the free blood *C*_max_ was 195 ng mL^–1^ and the concentration was above the EC_90_ for approximately 5 h. Assuming dose linearity, extrapolating the data to 100 mg kg^–1^ would provide free drug coverage above the EC_90_ for over 8 h. Since penetration and fraction unbound in the brain were not determined in rats, it is unclear whether the compound was above the free brain EC_90_ for any significant time. Thus, the efficacy of compound **38** was assessed in both mouse and rat models of stage 1 infection. Unfortunately, no reduction in parasitemia was observed in either infected rats (single dose, 100 mg kg^–1^ PO) or mice (*bid* 4 days 100 mg kg^–1^, NMRI mice). This could in part be due to the compound not achieving continual levels above the free EC_90_ in blood, a criterion which is generally desired.

### Assessing Cidality

To complement our established *T. brucei* cell-based screen, in the course of these studies, we developed an assay that allows discrimination of compounds that are cytostatic from those that are cytocidal.^15^ In this assay, static and cidal compounds are categorized by analysis of growth curves, with cidal compounds causing a decrease in parasite numbers. Commonly, phenotypic screens with *T. brucei* are end-point assays with live-cell indicators used as the final readout. In our reconfigured assay, bloodstream trypanosomes are grown in the presence of test compounds for 3 days with a starting cell density well below the assay limit of detection, thus making it impossible to distinguish cidal from static compounds. To assess if compounds within this diaminothiazole series kill trypanosomes or merely stop parasite growth, we profiled a panel of compounds in our static–cidal assay. Assessment of compound **38** in this assay demonstrated that only 50 μM led to a reduction in parasite number, suggesting that this compound only elicits a cytostatic effect below this concentration (referred to as “DDU1” in ref (15)). Based on our PK data, dosing mice with compound **38** at 100 mg kg^–1^ IP gave a free *C*_max_ in the blood of 16 μM, which is below the level required for a cidal effect, explaining the lack of activity in the mouse model of infection, even for peripheral disease. Given the extrapolated free *C*_max_ in the brain of 4.6 μM, the compound would likely have no efficacy in CNS infection either (not performed). Similarly in rats, the concentration would be below the level for cidal activity.

In contrast, the static–cidal assay enabled us to confirm that concentrations of compound **16** above 5.6 μM were cidal, while lower concentrations were cytostatic, perhaps suggesting that, at higher concentrations, **16** interacts with additional molecular targets that drive cidality (Figure S2). When compound **16** was dosed IP at 10 mg kg^–1^, it reached a *C*_max_ of 830 ng mL^–1^ (2.5 μM) in NMRI mice, likely insufficient for cidality. However, compound **16** was efficacious at 10 mg kg^–1^*bid* for 4 days. In HRN mice dosed in a similar manner, the *C*_max_ reached 3800 ng mL^–1^ (11 μM) and thus should have been sufficient to be cidal, but only one-third of the mice were cured. Collectively, these data suggest that the efficacy of compound **16** may be due to an active metabolite, produced in NMRI mice but not in HRN mice.

### Mode-of-Action Studies

2,4-Diaminothiazoles have long been associated with a variety of kinase targets.^13,14,16,17^ As previously described, this series diverged away from *Tb*GSK3 as a primary target.^7^ Here, we employed a range of unbiased approaches to determine the molecular target(s) of our lead compound **38**.

### Genome-Wide RNAi and Overexpression Screens

**Figure 3:**
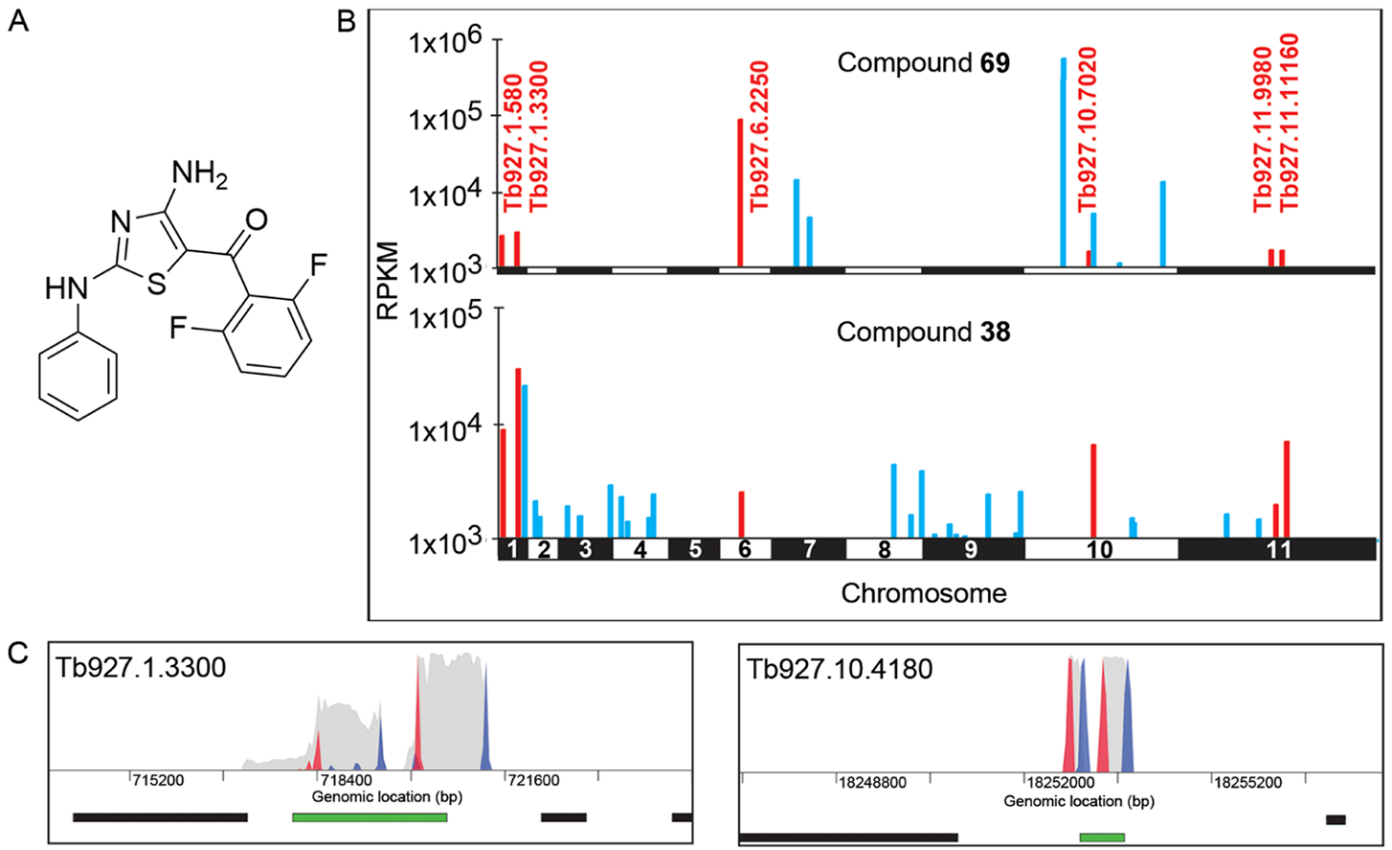
Genome-wide RNAi library screens. (A) Chemical structure of compound **69**. (B) Compounds **38** and **69** were screened against the *T. brucei* genome-wide RNAi library equivalent to 2× their established EC_50_ (600 and 300 nM for **38** and **69**, respectively). Genome-wide maps showing hits from each screen are shown. Several hits shared between compounds **38** and **69** (*T. brucei* gene IDs shown in red). RPKM: reads per kilobase of transcript per million mapped reads. See also Tables S3 and S4. (C) Focus on the top hits (individual genes) from RNAi screens with **38** (left; Tb927.1.3300) and **69** (right; Tb927.10.4180). Genes of interest are highlighted in green and other protein-coding regions in black. Red and blue peaks are RNAi construct forward and reverse barcodes, respectively. Gray peaks are all other reads.

Our genome-wide RNA interference (RNAi) library has proven invaluable in supporting our mode-of-action studies in *T. brucei*.^18−20^ While screening of the RNAi library does not directly identify the molecular targets of active compounds, it can be useful in identifying pathways and aspects of metabolism linked to compound action. During screening under tetracycline induction, each trypanosome within the library produces a unique double-stranded RNA (dsRNA) from an integrated RNAi fragment. The resulting interfering RNAs act to knock down the levels of specific targets with the target knockdown having the potential to confer a growth advantage under drug selection. Following selection, resistant trypanosomes are subjected to RNAi target sequencing (RIT-seq) to identify the specific RNAi fragments responsible for resistance.^21^ The RNAi library was screened with lead compound **38** and compound **69**, previously confirmed as an inhibitor of *Tb*GSK3^7^ (Figure 3A,B). Screens with both compounds, at concentrations equivalent to 2× their established EC_50_ values (600 and 300 nM for **38** and **69**, respectively), identified several distinct as well as overlapping “hits” (Figure 3B; Tables S3 and S4). Compounds acting via identical mechanisms of action would be expected to generate identical hits following library screening; thus, these shared and divergent “hits” illustrate the common origins of these compounds and suggest that their mechanisms of action have now diverged.

In keeping with our assumption that compounds from this series are likely to cause modulation of phosphorylation states, the top “hit” associated with compound **38** resistance was a phosphatase, specifically myotubularin-related phosphatase (Tb927.1.3300; Figure 3C). The MetaCyc database of metabolic pathways predicts that this phosphatase is involved in regulation of the inositol pyrophosphate pathway.^22^ The association of depleted levels of this phosphatase with resistance to compound **38** may implicate a corresponding kinase within the inositol pyrophosphate pathway as the molecular target of this diaminothiazole. Screening of the RNAi library with compound **69**, believed to target GSK3, identified the top hit as a “TFIIF-stimulated C-terminal domain (CTD) phosphatase” (Tb927.10.4180, Figure 3C). Domain analysis suggests this is a serine phosphatase that contains an FCP1 (TFIIF-associated CTD phosphatase) homology domain.^23^ Human FCP1 has been shown to interact with the transcription factor TFIIF and dephosphorylate the CTD of RNA polymerase II.^24,25^ Since human GSK3 is an RNA polymerase II phospho-CTD kinase, it is plausible that to bypass GSK3 inhibition, phosphorylated substrates (RNA polymerase II) are stabilized by knockdown of the corresponding phosphatase (TFIIF-stimulated CTD phosphatase).^26^ Further work beyond the scope of this study would be required to investigate this association. Both compound **38** and **69** RNAi library screens shared a number of “hits” (summarized in Tables S3 and S4). Several of these hits were associated with phosphate transport and phosphatase activity (Figure 3).

**Figure 4:**
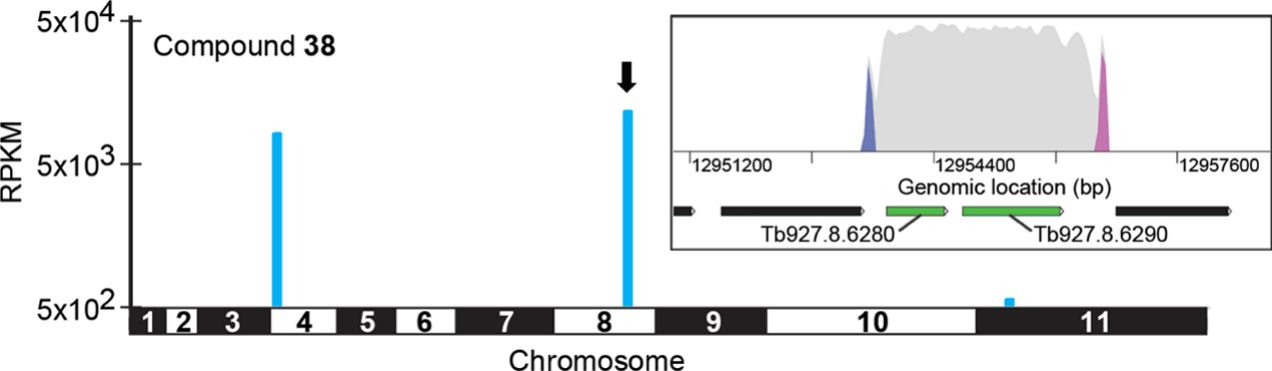
Genome-wide overexpression library screen with compound **38**. Genome-wide map showing the main hits are shown. RPKM: reads per kilobase of transcript per million mapped reads. The inset focuses on the top fragment hit of the overexpression (OE) library screen containing two full ORFs (Tb927.8.6280 and Tb927.8.6290). Genes of interest are highlighted in green and other protein-coding regions in black. Blue and pink peaks are OE construct forward and reverse barcodes (in the sense orientation), respectively. Gray peaks are all other reads. See also Table S5.

We next screened compound **38** against our *T. brucei* genome-wide overexpression library. This tetracycline-inducible library consists of trypanosomes each overexpressing a different protein, with expression driven by an RRNA promotor. The library is exposed to test compounds at concentrations equivalent to 2× their established EC_50_ values, with parasites capable of resisting this drug pressure sequenced to identify the overexpressed targets responsible for this resistance phenotype.^27^ Selection of the library with compound **38** enriched parasites bearing a genomic fragment containing neighboring open reading frames (ORFs) as the top “hit”: cyclophilin-type peptidyl-prolyl cis–trans isomerase (Tb927.8.6280) and a hypothetical protein (Tb927.8.6290) (Figure 4 and Table S5). This data indicates that overexpression of one of these proteins plays a significant role in resistance to compound **38**.

### Resistant Cell Line Generation Followed by Whole Genome Sequencing

**Figure 5:**
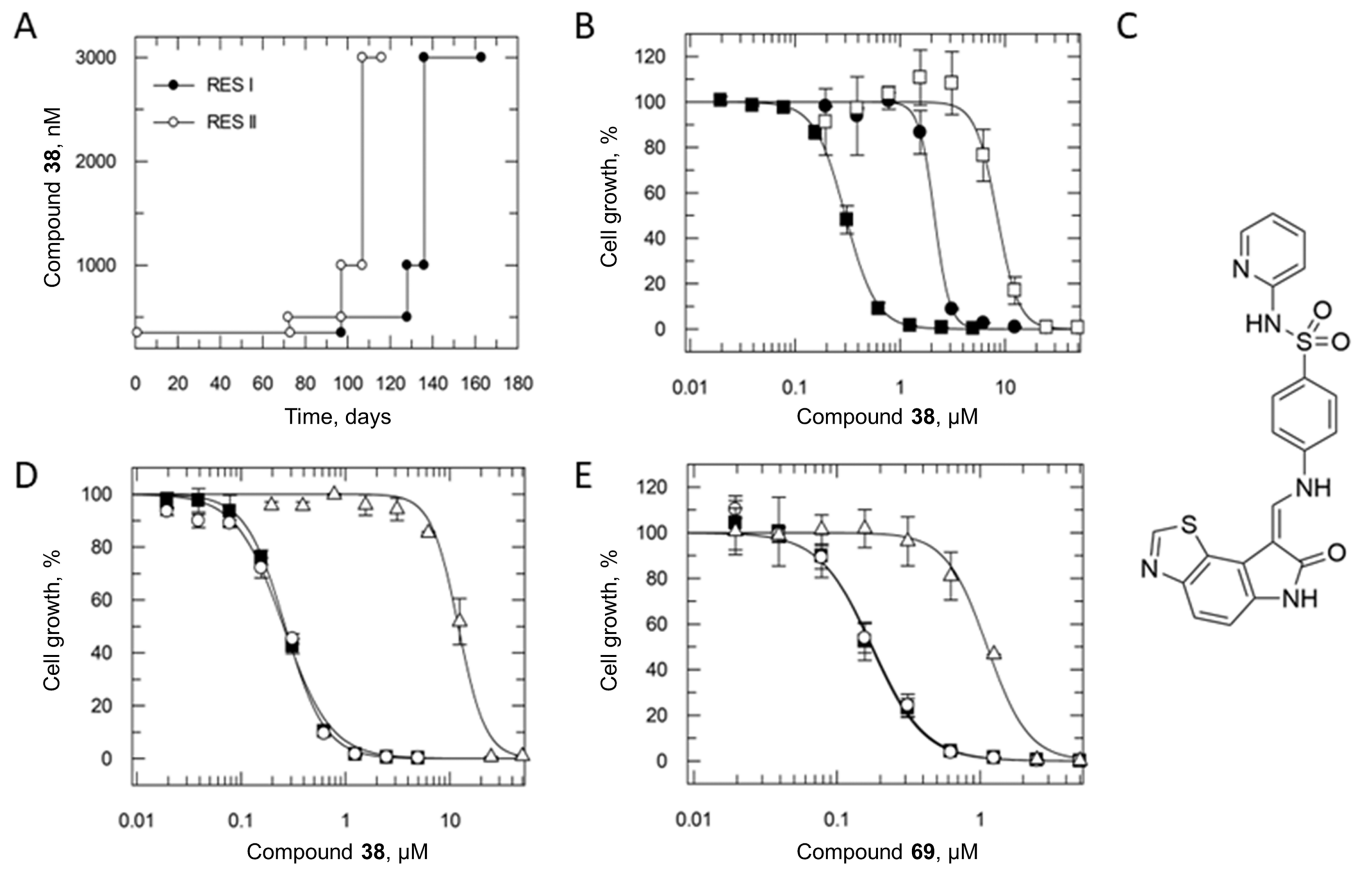
In vitro resistance generation against compound **38**. (A) Schematic showing the generation of compound **38**-resistant parasites. (B) Dose–response curves with compound **38** against wild-type (solid squares), RES I (solid circles), and RES II (open squares) parasites. Representative dose–response curves shown and weighed means are summarized in Table 5. Resistant parasites are 7–26-fold resistant to compound **38** compared with wild-type. (C) Chemical structure of GW8510. Dose–response curve of (D) compound **38** and (E) compound **69** with overexpression lines containing two mutations (Val241Phe and Ala258Val) observed in RES II. Overexpression lines were tested in the absence (presence and absence of 1 μg mL^–1^) of tetracycline: wild type (solid squares), −TET (open circles), and +TET (open triangles). Representative dose–response curves shown, and weighed means are shown in Table 6.

In a parallel approach to determine the molecular target(s) of compound **38**, parasites resistant to this diaminothiazole were generated by in vitro evolution. Two independent cultures of drug-sensitive, clonal trypanosomes were exposed to stepwise increasing concentrations of compound **38** over a 6 month period (Figure 5A). At this point, parasites were able to grow unhindered at concentrations of compound **38** in excess of 10-fold its established EC_50_ value. Following cloning by serial dilution, two independent clones (RES I and RES II) were selected for further study. These clones were between 7- and 26-fold less sensitive to compound **38** than the original parental wild-type (Figure 5B and Table 5). *Tb*GSK3 inhibitor **69** was also screened against the **38**-resistant lines. These clones were cross-resistant to **69**, albeit at a more modest level than compound **38**, in keeping with **38** and **69** having a partially shared mechanism of resistance/action (Table 5). In contrast, our clones were not cross-resistant to the structurally unrelated *T. brucei* GSK3 inhibitor (GW8510; Table 5 and Figure 5C^28^), suggesting that the mechanism(s) of resistance employed by these parasites does not specifically relate to GSK3 (Table 5).

**Table 5:** EC_50_ Data for Wild-Type and Resistant Cell Lines^a^

cell line	compound, nM		
	**38**	**69**	**GW8510**
wild-type	310 ± 4	150 ± 3	120 ± 8
RES I	2200 ± 84 (7)	360 ± 16 (2)	65 ± 4 (1)
RES II	8200 ± 490 (26)	390 ± 28 (3)	110 ± 4 (1)

a Values (nM) are the weighted mean of ≥3 independent experiments each consisting of two technical replicates. Fold resistance relative to the parental wild-type is shown in brackets.

Genomic DNA was isolated from compound **38**-resistant clones and analyzed by whole-genome sequencing. Several single-nucleotide polymorphisms (SNPs) were identified from both resistant lines on the same hypothetical protein (Tb927.8.6290) which was identified as a high-confidence “hit” in our overexpression library screen with compound **38** (Table S5). Specifically, RES I maintained a homozygous mutation (Ala258Pro), with RES II bearing two heterozygous mutations (Val241Phe and Ala258Val; both on the same allele). In addition, both resistant lines had a mutation encoding a premature stop codon on the gene encoding myotubularin-related phosphatase (Tb927.1.3300), essentially knocking out a copy of this gene. This phosphatase was identified as a high-confidence “hit” in our RNAi screens with compound **38**. Additional copy number variations and heterozygous mutations were identified and summarized (Table S6 and Figure S3); however, no changes specifically related to GSK3 (Tb427.10.13780) were identified.

### Target Validation

To investigate the role of the protein encoded by Tb927.8.6290 in the mode of action of these diaminothiazoles, clonal parasites overexpressing this hypothetical protein were generated. Label-free quantitative proteomics confirmed that once overexpression was induced, parasites maintained higher levels of this hypothetical protein than the wild-type (Figure S4). Overexpression led to a concomitant decrease (26-fold) in the potency of compound **38** compared to uninduced cells, essentially validating the results of our overexpression library (Table 6). We next generated clonal cell lines overexpressing the hypothetical protein (8.6290) bearing the mutations identified in our resistant cell lines (A258P and V241F/A258V). Once again, overexpression was confirmed by label-free quantitative proteomics (Figure S4). Overexpressing the mutated version of this protein conferred enhanced resistance (40–45-fold) to compound **38**, confirming that these mutations, identified in RES I and II, are directly involved in the resistance to this diaminothiazole (Figure 5D and Table 6). Interestingly, all three transgenic cell lines were cross-resistant to compound **69**, albeit to a lesser extent than **38** (Figure 5E and Table 6).

**Table 6:** EC_50_ Values for Cell Lines Overexpressing the Protein Encoded by Tb927.8.6290^a^

compounds	Tb927.8.6290 overexpressing cell lines					
	WT		A258P		V241F/A258V	
	–TET	+TET	–TET	+TET	–TET	+TET
**38**	400 ± 25	10000 ± 250 (26)	310 ± 9	12000 ± 250 (40)	270 ± 18	12000 ± 690 (45)
**69**	170 ± 4	410 ± 19 (2)	200 ± 9	1400 ± 35 (7)	180 ± 11	1200 ± 51 (6)

a EC_50_ values (nM) are the weighted mean ± SD of ≥2 independent experiments, each consisting of two technical replicates. Overexpression lines were tested in the absence (−TET) and presence (+TET) of 1 μg mL^–1^ tetracycline. Fold shift (shown in brackets) is calculated based on the difference between −TET and +TET EC_50_ values.

Based on this cross-resistance, we decided to further investigate the mechanism of action of **69** by screening this compound against our genome-wide overexpression library. Previous studies have demonstrated that overexpression of GSK3 is toxic for bloodstream trypanosomes; thus, we did not expect to identify this kinase as a “hit” in the screen.^29^ However, the screen did enrich parasites overexpressing the same hypothetical protein (encoded by Tb927.8.6290) identified as a top “hit” in the screen with compound **38** (Figure S5 and Table S7). These data, alongside the lack of cross-resistance demonstrated by the GSK3 inhibitor GW8510, suggest that the original *Tb*GSK3 inhibitor series exemplified by compound **69** likely had activity against multiple kinases and was not specific for *Tb*GSK3.

### Tb927.8.6290 Encodes a Homolog of Human ITPK1

**Figure 6:**
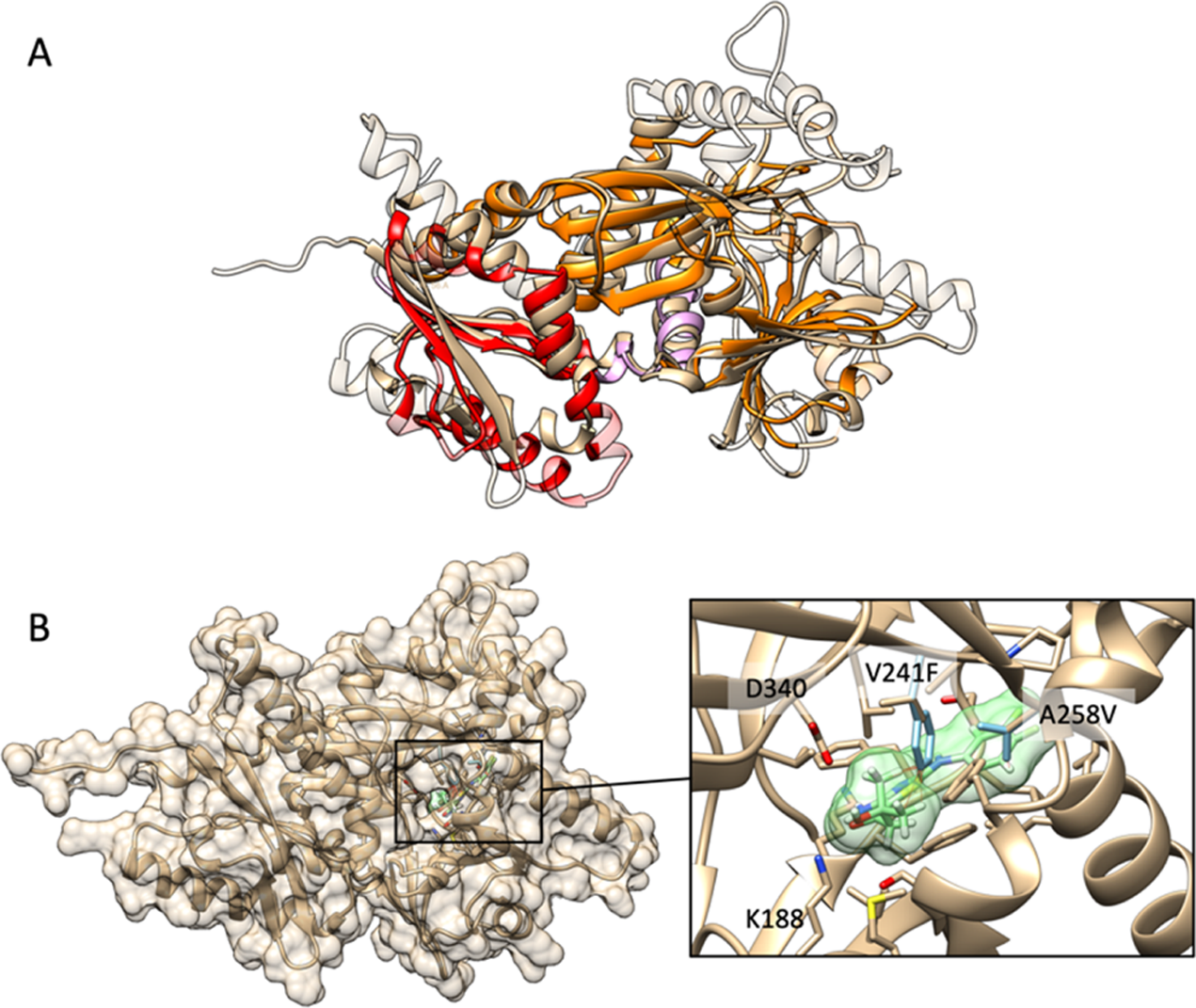
Structural model of “hypothetical protein” Tb927.8.6290 and mechanism of resistant mutants. (A) TriTrypAF model of Tb927.8.6290 (tan) superimposed with human inositol-tetrakisphosphate 1-kinase (ITPK1; PDB ID: 2qb5) (chain B).^37^ Overall rmsd = 2.3 Å. The human homolog is colored by its domains: ITPK1 N-terminus (red) and ATP-grasp domain (orange). See Figure S6 for domain-focused superpositions. Nonstructurally aligning regions are rendered transparent. (B) Model of resistance double mutant RES II (V241F/A258V) with compound **38** overlaid in its wild-type docking pose (mutant residues shown in light blue). See Figure S7 for models of both RES I and II with **38**, **69**, and ADP docked.

A protein domain search revealed that the hypothetical protein encoded by Tb927.8.6290 shares structural (but not sequence) similarity to inositol-tetrakisphosphate 1-kinase (ITPK1). Indeed, structure-based searches of the Protein Data Bank (PDB) using a predicted structural model of Tb927.8.6290 yielded human inositol-tetrakisphosphate 1-kinase (ITPK1) as the top-ranked hit.^30−33^ An additional search of the AlphaFold Protein Structure Database confirmed ITPK1 as the best candidate orthologue.^34^ Structural superposition of the putative *Tb*ITPK1 with ITPK1 illustrates significant overall structural similarity [root-mean-squared deviation (rmsd) = 2.3 Å], with the ATP-clasp domain particularly well conserved between proteins (Figures 6A and S6). These observations support the hypothesis that Tb927.8.6290 is a homolog of human ITPK1.

ITPK1 is a member of the inositol pyrophosphate pathway (also known as the phosphoinositide regulatory network). This complex cellular regulatory network consists of two major branches: phosphatidylinositol enzymes forming lipid-conjugated metabolites and inositol phosphate enzymes forming soluble metabolites. ITPK1 functions downstream of PI(4,5)P2, the main substrate for the second stage (inositol phosphate) of the pathway. Kinases of the inositol phosphate pathway are responsible for phosphorylation of the inositol ring to generate more complex inositol polyphosphates. For instance, classical ITPK1 kinases have both inositol-1,3,4-trisphosphate 5 and 6-kinase activities.^35^ ITPK1 has also been proposed to phosphorylate IP1 (inositol 1-phosphate and inositol 3-phosphate), enabling a lipid-independent route to generate inositol polyphosphates and for reversible phosphorylation of I(3,4,5,6)P4; thus, the exact function of this enzyme in *T. brucei* is unclear.^32,36^ To date, phylogenetic studies have failed to identify an ITPK1 orthologue in *T. brucei* perhaps due to limited sequence homology with the human enzyme.^29,33^

### Molecular Modeling and Docking Studies

To investigate the mechanism by which mutations in *Tb*ITPK1 confer resistance, compounds **38** and **69** were docked into the structural model of the putative kinase. Both compounds were predicted to bind to a region overlapping the ATP binding site and extend distally from the inositol phosphate pocket (Figures 6B and S7). All mutations are associated with resistance clusters around the predicted compound binding site. In RES II, the V241F mutation is likely to result in a steric clash with the thiazole sulfur moiety of both compounds and additionally with the R_2_ isobutyl alcohol of compound **38**. The A258V mutation results in a clash with the 3,4-difluorophenyl ring of **38** and the phenyl ring of **69**. While the A258P mutation of RES I is not predicted to cause steric clashes with bound compounds, analysis of the effect of the mutation on helix rigidity using DynaMut suggests that substantial dynamic effects occur in the vicinity of the compound binding sites (Figure S8;^38^). This could prevent the binding of **38** and **69** but not ATP. Based on our DynaMut analysis, the heterozygous mutations observed in RES II are likely to impact ATP binding to *Tb*ITPK1, thus affecting the function of this enzyme. However, the homozygous A258P mutation in RES I is not predicted to impact ADP/ATP binding (Figure S8). Furthermore, the additional clashes between the phenylalanine at position 241 with the isobutyl alcohol of **38** correlate with the enhanced resistance of RES II compared to RES I, while the absence of steric strain with the 2,6-difluorophenyl of compound **69** may account for the near identical EC_50_ values for this compound against RES I and RES II.

Our RNAi library and in vitro-generated resistant cell lines associate depletion of a myotubularin-related phosphatase (Tb927.1.3300) with compound **38** resistance. A structure-based search reveals that myotubularin-related phosphatase shares similarities with myotubularin-related protein-2 (MTMR2; PDB ID: 1m7r), a member of the inositol pyrophosphate pathway.^39^ It is tempting to suggest that the structural similarity with MTMR2 implicates this phosphatase as a previously unidentified member of the pathway. Myotubularins are predicted to dephosphorylate PI(3)P to PI (phosphatidylinositol) or PI(3,5)P_2_ (phosphatidylinositol 3,5-bisphosphate) to PI(5)P (phosphatidylinositol 5-phosphate). These reactions occur in the first stage (phosphatidylinositol) of the pathway, and no phosphatase orthologues capable of carrying out these functions have been identified in *T. brucei*.^40^ Further work is required to understand the function of this enzyme and to fully understand its role in resistance to compound **38**.

Collating the data within the current study, we hypothesize that compound **38** inhibits ITPK1 (Tb927.8.6290) and that knockdown of the myotubularin-related phosphatase, which appears to function upstream of this step of the pathway, confers resistance by regulating lipid-conjugated metabolite levels.

The inositol pyrophosphate pathway produces phosphorylated derivatives of myoinositol that are involved in the regulation of multiple cellular processes. Many of the enzymes of this pathway are essential in *T. brucei*.^41,42^ However, these studies have confirmed that treatment with compound **38** elicits a cytostatic effect. Entirely in keeping with ITPK1 as the target of this compound, genome-wide loss-of-fitness screens in *T. brucei* confirm that Tb927.8.6290 is not essential for cell viability but that knockdown does significantly impact cell growth.^21^ Further investigation of the functional role(s) of these proteins (Tb927.1.3300 and Tb927.8.6290) in the inositol pyrophosphate pathway is merited.

## Conclusions

Here, we describe the optimization of a phenotypic hit derived from a target-based project. The project encountered the typical challenges of a drug discovery program, with multiparametric optimization required, for potency, selectivity, microsomal stability, solubility, and blood–brain barrier permeability. Excellent potency was achieved against bloodstream trypanosomes in cell-based assays, and the compound series displayed good pharmacokinetic properties including blood–brain barrier penetration, an essential feature for any new therapeutic to treat stage 2 HAT. In addition, by introducing amines and ether substituents to the scaffold, good solubility levels were achieved.

This study highlights the need for cytocidal drugs for the effective treatment of HAT. Most standard cell-based assays, used to assess potency, are not capable of distinguishing between cidal and static compounds. During optimization of the pharmacokinetic properties, compounds in this series evolved from efficacious in a mouse model to nonefficacious, despite retaining similar levels of potency in cell-based assays. Our bespoke static–cidal assay confirms that during optimization, the series progressed from cytocidal compounds capable of in vivo activity to a series where the main mode of action was cytostatic, with cidality only apparent at much higher concentrations, which were unobtainable in a mouse model of infection. The comprehensive mechanism of action studies provides a molecular basis for the cytostatic nature of compounds in this series. A range of unbiased approaches suggest that compounds from this series target a kinase (putative *Tb*ITPK1) that may form part of the inositol pyrophosphate pathway. This putative target is not essential for *T. brucei* viability, thus explaining the cytostatic phenotype elicited by compounds that target this kinase. It should also be noted that PK exposures for compound **38**, determined in both rat and mouse models, are unlikely to be good enough for efficacy irrespective of the static versus cidal target switch. Clearly, improving exposure would have been a key aspect of future studies with this series had this target switch not occurred.

Based on the experience gained in this study, static–cidal assays are now a fundamental part of our kinetoplastid drug discovery workflow, especially for phenotypic series. Routine screening of analogues throughout the evolution of series provides confidence that compounds remain cidal and prevents wasting valuable time and resources.

## Experimental Section

### Chemistry

#### Purity

All compounds reported in this study were >95% pure as determined by LC–MS. See details in general methods (Supporting Information).

### Chemical Synthesis

#### α-Bromoketones

Noncommercially available α-bromoketones were synthesized from either the corresponding acid chloride or where this was unavailable, the corresponding acid (Scheme S1). Where necessary, the required acid was heated in thionyl chloride to afford its acid chloride equivalent. The relevant acid chloride was reacted with trimethylsilyldiazomethane to afford the diazoketone equivalent, and in situ reaction with hydrobromic acid gave the desired α-bromoketones (Scheme S1).^4^

### General Procedures

General procedures A–E used in the syntheses described below can be found in the Supporting Information.

#### 2,4-Diamino-5-ketothiazoles

Noncommercially available isothiocyanates were prepared as outlined in Scheme S2 (step a). R_1_ amine was reacted with thiocarbonyl diimidazole to afford its corresponding isothiocyanate **5**. A one-pot two-step cyclization was employed to yield the 2,4-diamino-5-ketothiazoles (Scheme S2); reaction with either benzyl carbaminidothioate hydrobromide salt **6a** or 3,5-dimethyl-1*H*-pyrazole-1-carboximidamide nitrate salt **6b** formed stable thiourea that reacted in situ with a range of α-bromoketones to afford the 2,4-diamino-5-ketothiazoles **7** (see ref (5) and references therein).

For the synthesis of 2,4-diaminothiazole-5-(2-etherethanones), **9**, and 2,4-diaminothiazole-5-(2-aminoethanones), **10**, a common 2,4-diaminothiazole-5-(2-bromoethanone), **8**, intermediate was synthesized using the route described in Scheme S2 and 1,3-dibromopropan-2-one. The ether compounds were prepared using sodium hydride and substituted alcohols (Scheme S3, route b), and the amino compounds were prepared by direct displacement of the bromo with substituted amines (Scheme S3, route c).

#### 1-Chloro-3-((1,1,1-trifluoropropan-2-yl)oxy)propan-2-one Intermediate

For array synthesis where the 5-substituent on the thiazole was 2-((1,1,1-trifluoropropan-2-yl)oxy)ethanone, a stock intermediate of 1-chloro-3-((1,1,1-trifluoropropan-2-yl)oxy)propan-2-one **14** was prepared from 2-bromoacetylbromide **11** (Scheme S4). **11** was reacted with *N*,*O*-dimethylhydroxylamine hydrochloride to give 2-bromo-*N*-methoxy-*N*-methylacetamide **12**; the reaction of **12** with sodium 1,1,1-trifluoropropan-2-olate gave *N*-methoxy-*N*-methyl-2-(2,2,2-trifluoroethoxy)acetamide **13**. The reaction of **13** with methyl lithium and chloroiodomethane generated (1-chloro-3-(1,1,1-trifluoropropan-2-yl)oxy)propan-2-one **14**. The reaction of various isothiocyanates with **14** as outlined in Scheme S2 produced a range of 2-((1,1,1-trifluoropropan-2-yl)oxy)ethanone substituted thiazoles **9**.

### Preparation of Standard Intermediates

#### Benzyl Carbaminidothioate Hydrobromide

Benzyl bromide (16.0 mL, 135 mmol), thiourea (10.0 g, 131 mmol), and EtOAc (75 mL) were combined and heated at 120 °C in a microwave for 5 min. The reaction mixture was cooled to rt and the resulting solid collected by filtration to afford the title compound as a white solid 29.3 g, 89% yield; δ_H_ (500 MHz, DMSO-*d*_6_): 9.07 (bs, 4H), 7.44–7.32 (m, 5H), 4.49 (s, 2H).

### Prototypical Procedure: Synthesis of α-Bromoketones from Acid Chlorides

#### 1,3-Dibromo-3-methylbutan-2-one

2-Bromo-2-methylpropanoyl chloride (17.7 mmol) in acetonitrile (anhydrous, 170 mL) was cooled to 0 °C, TMS–diazomethane (35.4 mmol) was added slowly, and the reaction mixture was stirred at 0 °C for 1 h. HBr (3.84 mL, 35.4 mmol) was added dropwise slowly at 0 °C. The mixture was stirred at rt for 10 min, quenched with a 1 M NaOH solution (75 mL), extracted into EtOAc, and washed with a sat. aq NaHCO_3_, H_2_O, and then brine, and the organic layer was dried over MgSO_4_. Crude ^1^H NMR indicated >98% purity for the desired product, which was used without further purification, 1.2 g, 28%; δ_H_ (500 MHz, CDCl_3_): δ 3.79 (s, 2H, CH_2_), 1.94 (s, 6H, 2 × CH_3_).

### Experimental Details for Analogues Detailed in Table 1

#### Prototypical Examples of General Procedure A (Supporting Information)

##### 1-(4-Amino-2-(cyclohexylamino)thiazol-5-yl)-2,2-dimethylpropan-1-one (**1**)

2-Benzylisothiouronium bromide (0.25 g, 1 mmol), DIPEA (0.19 mL, 1.1 mmol), DMF (1.5 mL mmol^–1^), cyclohexylisothiocyanate (0.14 mL, 1.05 mmol), 1-bromo-3,3-dimethylbutan-2-one (0.16 mL, 1.2 mmol), and DIPEA (0.35 mL, 2 mmol) were reacted as described in general procedure A to afford the title compound as a yellow powder 171 mg, 61% yield; δ_H_ (500 MHz, CDCl_3_): 5.34 (d, *J* = 7.1 Hz, 1H), 3.30–3.28 (m, 1H), 2.00 (dd, *J* = 13.6 and 3.3 Hz, 1H), 1.70 (dt, *J* = 13.6 and 4.0 Hz, 2H), 1.57 (dt, *J* = 13.2 and 4.0 Hz, 1H), 1.38–1.33 (m, 2H), 1.20 (s, 9H, ^*t*^Bu–H), 1.19–1.17 (m, 1H), 0.81 (t, *J* = 7.1 Hz, 1H), 0.79–0.76 (m, 1H). LCMS (ES+) *m*/*z*: (%) 282 [M + H]^+^*t*_R_ 4.60 (20–90% MeCN, acidic). HRMS (ES+) calcd for [C_14_H_23_N_3_OS + H], 282.1635; found, 282.1641.

##### 1-(4-Amino-2-(cyclohexylamino)thiazol-5-yl)-2-methoxy-2-methylpropan-1-one (**17**)

1-(4-Amino-2-(cyclohexylamino)thiazol-5-yl)-2-bromo-2-methylpropan-1-one (30 mg, 0.09 mmol) and NaO^*t*^Bu (17 mg, 0.18 mmol) were heated in MeOH (anhydrous, 2 mL) at 50 °C for 16 h, the excess solvent was removed, and the crude residue was partitioned between DCM and H_2_O. Column chromatography elution with petroleum ether (40–60 °C)/EtOAc (4:1) afforded the title compound as a colorless solid, 20 mg, 78%; δ_H_ (500 MHz, CDCl_3_): 5.46 (bs, 1H, NH), 3.40 (bs, 1H, CH), 3.26 (s, 3H, CH_3_), 2.11–2.09 (m, 2H, CH), 1.79 (dt, *J* = 13.7 and 3.7, 2H, CH), 1.66 (dt, *J* = 13.0 and 3.7 Hz, 1H, CH), 1.59 (s, 4H, CH), 1.42 (s, 6H, 2 × CH_3_), 1.32–1.25 (m, 3H, CH); LCMS (ES+) *m*/*z*: (%) 298 [M + H]^+^*t*_R_ 4.17 (20–90% MeCN, acidic); HRMS (ES+) calcd for [C_14_H_23_N_3_O_2_S + H], 298.1584; found, 298.1587.

##### 1-(4-Amino-2-(cyclohexylamino)thiazol-5-yl)-2-methylpropan-1-one (**18**)

Prepared following general procedure A, 112 mg, 42%; δ_H_ (500 MHz, CDCl_3_): 5.56 (bs, 1H, NH), 3.15 (bs, 1H, CH), 2.44 (sep, *J* = 6.8 Hz, 1H, CH), 1.89 (dd, *J* = 12.5 and 3.5 Hz, 2H, CH), 1.61 (dt, *J* = 13.6 and 3.9 Hz, 2H, CH), 1.49 (dt, *J* = 13.0 and 3.8 Hz, 1H, CH), 1.25 (ddt, *J* = 25.0, 11.7 and 3.5 Hz, 2H, CH), 1.14–1.04 (m, 3H, CH), 1.00 (s, 3H, CH_3_), 0.99 (s, 3H, CH_3_); δ_C_ (125 MHz, CDCl_3_): 193.7 (C=O), 170.0, 163.8, 93.4 (ArC), 54.9, 39.6, 32.8, 25.3, 24.6, 19.2; LCMS (ES+) *m*/*z*: (%) 268 [M + H]^+^*t*_R_ 4.37 (20–90% MeCN, basic); HRMS (ES+) calcd for [C_13_H_22_N_3_OS + H], 268.1478; found, 268.1466.

##### (4-Amino-2-(cyclohexylamino)thiazol-5-yl)cyclobutylmethanone (**19**)

Prepared following general procedure A, 148 mg, 53%; δ_H_ (500 MHz, DMSO-*d*_6_): 8.49 (bs, 1H), 7.74 (bs, 2H, NH_2_), 3.56 (bs, 1H), 3.18 (q, *J* = 8.1 Hz, 1H), 2.23–2.18 (m, 2H), 2.11–2.06 (m, 2H), 2.01–1.93 (m, 3H), 1.81–1.75 (m, 3H), 1.64–1.61 (m, 1H), 1.35–1.19 (m, 5H); LCMS (ES+) *m*/*z*: (%) 314 [M + H]^+^*t*_R_ 4.31 (20–90% MeCN, acidic).

##### (4-Amino-2-(cyclohexylamino)thiazol-5-yl) (cyclohexyl)methanone (**20**)

Prepared following general procedure A, 85 mg, 28%; δ_H_ (500 MHz, CDCl_3_): 5.99 (bs, 1H, NH), 3.32 (bs, 1H, CH), 2.32 (tt, *J* = 11.7 and 3.3 Hz, 1H, CH), 2.09–2.07 (m, 2H, CH), 1.73–1.66 (m, 2H, CH), 1.56 (dd, *J* = 12.3 and 2.9 Hz, 1H, CH), 1.51–1.49 (m, 1H, CH), 1.45 (dd, *J* = 11.6 and 3.4 Hz, 1H, CH), 1.40–1.38 (m, 1H, CH), 1.36–1.24 (m, 6H, CH); LCMS (ES+) *m*/*z*: (%) 308 [M + H]^+^*t*_R_ 5.1 (5–95% MeCN, basic).

##### (4-Amino-2-(cyclohexylamino)thiazol-5-yl)(2,6-dichlorophenyl)methanone (**21**)

Prepared following general procedure A, 140 mg, 38%; δ_H_ (500 MHz, CDCl_3_): 7.26 (d, *J* = 7.9 Hz, 2H, ArH), 7.19–7.17 (m, 2H, ArH), 5.61 (bs, 1H, NH), 3.14 (bs, 1H, NH), 1.94 (bd, *J* = 12.3 Hz, 2H, CH_3_), 1.67 (dt, *J* = 13.4 and 3.9 Hz, 2H, CH_2_), 1.27–1.11 (m, 5H, CH), 0.80–0.76 (m, 1H, CH); LCMS (ES+) *m*/*z*: (%) 372 and 370 ^35^Cl and ^37^Cl [M + H]^+^*t*_R_ 4.4–4.6 (20–90% MeCN, basic).

##### (4-Amino-2-(cyclohexylamino)thiazol-5-yl)(2,6-difluorophenyl)methanone (**16**)

Prepared following general procedure A, 179 mg, 53%; δ_H_ (500 MHz, CDCl_3_): 7.49–7.43 (m, 1H, ArH), 7.07 (dd, *J* = 8.4 and 7.2 Hz, 2H, ArH), 5.96 (bs, 1H, NH), 3.56 (bs, 1H, CH), 2.14 (dd, *J* = 12.5 and 3.1 Hz, 2H, CH), 1.87 (dt, *J* = 13.5 and 4.0 Hz, 2H, CH), 1.74 (dt, *J* = 13.1 and 4.0 Hz, 1H, CH), 1.51–1.31 (m, 5H, CH); δ_C_ (125 MHz, CDCl_3_): 174.3 (C=O), 172.4, 165.1, 158.3, 158.2, 130.7, 111.9, 98.2 (ArC), 55.1, 32.7, 25.2, 24.5; LCMS (ES+) *m*/*z*: (%) 338 [M + H]^+^*t*_R_ 4.7–4.8 (5–95% MeCN, basic); HRMS (ES+) calcd for [C_16_H_18_F_2_N_3_OS + H], 298.1584; found, 298.1587.

##### (4-Amino-2-(cyclohexylamino)thiazol-5-yl)(4-fluorophenyl)methanone (**22**)

Prepared following general procedure A, 78 mg, 24%; δ_H_ (500 MHz, CDCl_3_): 7.79–7.77 (m, 2H, ArH), 7.12 (t, *J* = 8.8 Hz, 1H, ArH), 5.56 (bs, 1H, NH), 3.33 (bs, 1H, CH), 2.08 (dd, *J* = 12.3 and 2.9 Hz, 2H, CH), 1.79 (dt, *J* = 13.8 and 4.1 Hz, 2H, CH), 1.66 (dt, *J* = 13.1 and 3.7 Hz, 1H, CH), 1.33–1.26 (m, 3H, CH), 1.43 (ddt, *J* = 25.0, 11.5 and 3.3 Hz, 2H, CH); LCMS (ES+) *m*/*z*: (%) 320 [M + H]^+^*t*_R_ 4.48 (20–90% MeCN, acidic); HRMS (ES+) calcd for [C_16_H_19_FN_3_OS + H], 320.1227; found, 320.1226.

##### (4-Amino-2-(cyclohexylamino)thiazol-5-yl)(4-(trifluoromethyl)phenyl)methanone (**23**)

Prepared following general procedure A, 110 mg, 30%; δ_H_ (500 MHz, CDCl_3_): 7.75 (d, *J* = 8.1 Hz, 2H, ArH), 7.61 (d, *J* = 8.1 Hz, 2H, ArH), 5.67 (d, *J* = 6.2 Hz, 1H, NH), 3.22 (bs, 1H, CH), 1.99–1.96 (m, 2H, CH), 1.69 (tt, *J* = 13.6 and 3.9 Hz, 2H, CH), 1.57 (tt, *J* = 13.0 and 3.8 Hz, 1H, CH), 1.29 (ddt, *J* = 25.0, 11.6 and 3.3 Hz, 2H, CH), 1.23–1.13 (m, 3H, CH); δ_C_ (125 MHz, CDCl_3_): 182.2 (C=O), 172.1, 166.1, 145.0, 127.5, 125.4, 125.4, 122.8 (ArC), 94.2, 55.2, 32.7, 25.2, 24.6; LCMS (ES+) *m*/*z*: (%) 370 [M + H]^+^*t*_R_ 4.58 (20–90% MeCN, acidic); HRMS (ES+) calcd for [C_17_H_18_F_3_N_3_OS + H], 370.1195; found, 370.1195.

##### (4-Amino-2-(cyclohexylamino)thiazol-5-yl)(*p*-tolyl)methanone (**24**)

Prepared following general procedure A, 238 mg, 76%; δ_H_ (500 MHz, CDCl_3_): 7.57 (d, *J* = 8.0 Hz, 2H, ArH), 7.16 (d, *J* = 8.0 Hz, 2H, ArH), 5.50 (d, *J* = 6.6 Hz, 1H, NH), 3.23 (bs, 1H, NH), 1.98 (dd, *J* = 12.2 and 2.7 Hz, 2H, CH), 1.68 (dt, *J* = 13.6 and 4.0 Hz, 2H, CH), 1.29 (ddt, *J* = 25.0, 11.5 and 3.1 Hz, 2H, CH), 1.23–1.13 (m, 3H, CH); LCMS (ES+) *m*/*z*: (%) 316 [M + H]^+^*t*_R_ 4.60 (20–90% MeCN, basic).

##### (4-Amino-2-(cyclohexylamino)thiazol-5-yl)(pyridine-4-yl)methanone (**25**)

Prepared following general procedure A, 39 mg, 13%; δ_H_ (500 MHz, CDCl_3_): 8.74 (dd, *J* = 4.4 and 1.7 Hz, 2H, py-H), 7.60 (dd, *J* = 4.4 and 1.7 Hz, 2H, py-H), 5.62 (bs, 1H, NH), 3.35 (bs, 1H, CH), 2.08 (dd, *J* = 13.0 and 3.8 Hz, 1H, CH), 1.80 (dt, *J* = 10.0 and 3.3 Hz, 2H, CH), 1.69–1.66 (m, 1H, CH), 1.43–1.37 (m, 2H, CH), 1.34–1.24 (m, 3H, 2 × CH); δ_C_ (125 MHz, CDCl_3_): 170.5 (C=O), 153.1, 152.6, 124.9 (ArC), 35.9, 28.6, 28.1; LCMS (ES+) *m*/*z*: (%) 303 [M + H]^+^*t*_R_ 3.30 (20–90% MeCN, basic); HRMS (ES+) calcd for [C_15_H_18_N_4_OS + H], 303.1274; found, 303.1284.

##### (4-Amino-2-(cyclohexylamino)thiazol-5-yl)(4-(difluoromethoxy)phenyl)methanone (**26**)

Prepared following general procedure A, 179 mg, 49%; δ_H_ (500 MHz, CDCl_3_): 7.69 (dd, *J* = 6.7 and 2.1 Hz, 2H, ArH), 7.09 (d, *J* = 8.9 Hz, 2H, ArH), 6.50 (t, *J* = 73.6 Hz) (fluorine splitting, OCHF_2_), 6.00 (bs, 1H, NH), 3.21 (bs, 1H, CH), 1.97 (dd, *J* = 12.6 and 3.2 Hz, 2H, CH), 1.70 (dt, *J* = 13.6 and 3.9 Hz, 2H, CH), 1.56 (dt, *J* = 12.9 and 3.9 Hz, 1H, CH), 1.30 (ddd, *J* = 25.0, 11.7 and 3.3 Hz, 2H, CH), 1.20–1.13 (m, 3H, CH); LCMS (ES+) *m*/*z*: (%) 368 [M + H]^+^*t*_R_ 4.50 (20–90% MeCN, acidic); HRMS (ES+) calcd for [C_17_H_20_F_2_N_3_O_2_S + H], 368.1239; found, 368.1241.

##### (4-Amino-2-(cyclohexylamino)thiazol-5-yl)(2-fluorophenyl)methanone (**27**)

Prepared following general procedure A, 220 mg, 69%; δ_H_ (500 MHz, CDCl_3_): 7.52 (td, *J* = 7.3 and 1.7 Hz, 1H, ArH), 7.43–7.38 (m, 1H, ArH), 7.21 (dt, *J* = 7.5 and 0.8 Hz, 1H, ArH), 7.16–7.12 (m, 1H, ArH), 5.86 (bs, 1H, NH), 3.26 (bs, 1H, CH), 2.03 (dd, *J* = 12.5 and 3.2 Hz, 2H, CH), 1.76 (dt, *J* = 13.4 and 4.0 Hz, 2H, CH), 1.64 (dt, *J* = 12.8 and 3.7 Hz, 1H, CH), 1.38–1.21 (m, 5H, CH); LCMS (ES+) *m*/*z*: (%) 320 [M + H]^+^*t*_R_ 4.29 (20–90% MeCN, acidic).

##### (4-Amino-2-(cyclohexylamino)thiazol-5-yl)(2,4-difluorophenyl)methanone (**29**)

Prepared following general procedure A, 102 mg, 30%; δ_H_ (500 MHz, CDCl_3_): 7.44–7.40 (m, 1H, ArH), 6.90–6.78 (m, 2H, ArH), 3.14 (bs, 1H, CH), 1.96–1.94 (m, 3H, CH_2_), 1.69 (dt, *J* = 13.3 and 4.0 Hz, 2H, CH_2_), 1.57–1.52 (m, 1H, CH_2_), 1.32–1.20 (m, 4H, CH_2_); LCMS (ES+) *m*/*z*: (%) 338 [M + H]^+^*t*_R_ 4.41 (20–90% MeCN, acidic); HRMS (ES+) calcd for [C_16_H_17_F_2_N_3_OS + H], 338.1133; found, 338.1118.

##### (4-Amino-2-(cyclohexylamino)thiazol-5-yl)(3,4-difluorophenyl)methanone (**30**)

Prepared following general procedure A, 121 mg, 36%; δ_H_ (500 MHz, CDCl_3_): 1 proton masked under CDCl_3_ peak, 7.24 (s, 1H, ArH), 6.93 (tt, *J* = 8.7 and 2.2 Hz, 1H, ArH), 5.69 (bs, 1H, NH), 3.36 (bs, 1H, CH), 2.09 (dd, *J* = 13.4 and 3.8 Hz, 2H, CH), 1.81 (dt, *J* = 13.3 and 3.9 Hz, 2H, CH), 1.69 (dt, *J* = 13.5 and 4.2 Hz, 1H, CH), 1.46 (ddt, *J* = 25.0, 11.7 and 3.3 Hz, 2H, CH), 1.35–1.25 (m, 3H, 3 × CH); δ_C_ (125 MHz, CDCl_3_): 181.0 (C=O), 171.9, 168.3, 165.5, 150.6, 138.5, 129.0, 123.7, 117.1, 93.3, 55.4, 32.6, 25.2, 24.5; LCMS (ES+) *m*/*z*: (%) 338 [M + H]^+^*t*_R_ 4.55 (20–90% MeCN, acidic); HRMS (ES+) calcd for [C_16_H_17_F_2_N_3_OS + H], 338.1133; found, 338.1131.

##### (4-Amino-2-(cyclohexylamino)thiazol-5-yl)(3,5-difluorophenyl)methanone (**31**)

Prepared following general procedure A, 156 mg, 46%; δ_H_ (500 MHz, CDCl_3_): 7.51 (ddd, *J* = 10.8, 7.7 and 2.1 Hz, 1H, ArH), 7.44 (ddd, *J* = 8.4, 4.2 and 1.5 Hz, 1H, ArH), 7.13 (dd, *J* = 18.1 and 8.1 Hz, 1H, ArH), 5.75 (bs, 1H, NH), 3.23 (bs, 1H, CH), 1.98 (dd, *J* = 12.2 and 3.2, 2H, CH), 1.71 (dt, *J* = 13.7 and 4.0 Hz, 1H, CH), 1.58 (dt, *J* = 12.9 and 4.0 Hz, 1H, CH), 1.36–1.28 (m, 2H, CH), 1.25–1.13 (m, 3H, CH); δ_C_ (125 MHz, CDCl_3_): 180.6 (C=O), 171.9, 166.3, 163.7, 161.7, 144.8, 110.4, 110.3, 105.6, 93.8, 55.1, 32.8, 25.2, 24.5; LCMS (ES+) *m*/*z*: (%) 338 [M + H]^+^*t*_R_ 4.57 (20–90% MeCN, acidic); HRMS (ES+) calcd for [C_16_H_17_F_2_N_3_OS + H], 338.1133; found, 338.1138.

##### Cyclohexyl(2-(cyclohexylamino)thiazole-5-yl)methanone (**32**)

*N*′-(Cyclohexylcarbamothionyl)-*N*,*N*-dimethylformimidamide (107 mg, 0.5 mmol), 2-bromo-1-cyclohexylethanone (102 mg, 0.6 mmol), and TEA (0.21 mL, 1.5 mmol) were heated in ethanol (4 mL) for 16 h and cooled to rt; the solvent was removed in vacuo, and the crude residue was purified by column chromatography, eluting with petroleum ether (40–60 °C)/EtOAc (4:1) to afford the desired product as a colorless solid, 57 mg, 39%; δ_H_ (500 MHz, CDCl_3_): 7.71 (s, 1H, thiazole-H), 5.99 (bs, 1H, NH), 3.29 (bs, 1H, CH), 2.86 (tt, *J* = 11.7 and 3.1 Hz, 1H, CH), 2.01 (dd, *J* = 12.2 and 2.6 Hz, 2H, CH), 1.77 (d, *J* = 10.6 Hz, 2H, CH), 1.71 (tt, *J* = 13.5 and 4.0 Hz, 2H, CH), 1.64 (d, *J* = 12.6 Hz, 2H, CH), 1.58 (dt, *J* = 13.1 and 4.0 Hz, 2H, CH), 1.51–1.41 (m, 2H, CH), 1.35–1.17 (m, 8H, CH); LCMS (ES+) *m*/*z*: (%) 293 [M + H]^+^*t*_R_ 4.74 (20–95% MeCN, acidic); HRMS calcd for [C_16_H_25_N_2_OS + H], 293.1682; found, 293.1682.

##### (2-(Cyclohexylamino)thiazol-5-yl)(2,6-difluorophenyl)methanone (**33**)

*N*′-(Cyclohexylcarbamothionyl)-*N*,*N*-dimethylformimidamide (107 mg, 0.5 mmol), 2-bromo-1-(2,6-difluorophenyl)ethanone (120 mg, 0.6 mmol), and TEA (0.21 mL, 1.5 mmol) were heated in ethanol (4 mL) for 16 h and cooled to rt; the solvent was removed in vacuo, and the crude residue was purified by column chromatography, eluting with petroleum ether (40—60 °C)/EtOAc (4:1) to afford the desired product as a colorless solid, 152 mg, 94%; δ_H_ (500 MHz, CDCl_3_): 7.42 (s, 1H, ArH), 7.37–7.31 (m, 1H, ArH), 6.92 (dd, *J* = 8.4 and 7.2 Hz, 2H, ArH), 6.26 (bs, 1H, NH), 3.32 (bs, 1H, CH), 2.03 (dd, *J* = 12.5 and 2.9 Hz, 2H, CH), 1.72 (dt, *J* = 13.5 and 4.0 Hz, 2H, CH), 1.59 (dt, *J* = 12.9 and 3.9 Hz, 1H, CH), 1.38–1.16 (m, 5H, CH); LCMS (ES+) *m*/*z*: (%) 323 [M + H]^+^*t*_R_ 4.57 (20–95% MeCN, acidic); HRMS calcd for [C_16_H_17_F_2_N_2_OS + H], 323.1024; found, 323.1013.

##### (4-Amino-2-(phenylamino)thiazol-5-yl)(phenyl)methanone (**34**)

Prepared following general procedure A, yellow solid, 47 mg, 39% yield; δ_H_ (500 MHz, DMSO-*d*_6_): 10.80 (bs, 1H, NH), 8.22 (bs, 2H, NH_2_), 7.69–7.67 (m, 2H, PhH), 7.62 (d*, J* = 7.7 Hz, 2H, PhH), 7.51–7.46 (m, 3H, PhH), 7.39–7.36 (m, 2H, PhH), 7.09 (tt, *J* = 7.4 and 1.0 Hz, 1H, PhH). LCMS (ES+) *m*/*z*: (%) 296 [M + H]^+^*t*_R_ 4.28 (20–95% MeCN, acidic).

##### (4-Amino-2-(phenylamino)thiazol-5-yl)(4-fluorophenyl)methanone (**35**)

Prepared following general procedure A, 103 mg, 33%; δ_H_ (500 MHz, DMSO-*d*_6_): 10.83 (s, 1H), 8.23 (bs, 2H), 7.77–7.73 (m, 2H), 7.62 (d, *J* = 7.7 *Hz*, 2H), 7.39–7.29 (m, 4H), 7.10 (tt, *J* = 7.4 and 1.0 *Hz*, 1H); LCMS (ES+) *m*/*z*: (%) 346 [M + H]^+^*t*_R_ 4.60 (20–90% MeCN, acidic).

##### 1-(4-Amino-2-((4-fluorophenyl)amino)thiazol-5-yl)-3-methylbutan-1-one (**36**)

Prepared following general procedure A, 40 mg, 41%; δ_H_ (500 MHz, DMSO-*d*_6_): 10.70 (s, 1H, NH), 7.79 (br s, 2H, NH_2_), 7.63 (m, 2H, ArH), 7.21 (m, 2H, ArH), 2.21 (bd, *J* = 7.0 Hz, 2H), 2.09 (sept, *J* = 7.0 Hz, 1H), 0.90 (d, *J* = 7.0 Hz, 6H, 2 × CH_3_); HRMS (ES+) calcd for [C_14_H_16_FN_3_OS + H], 294.1063; found, 294.1071.

##### 1-(4-Amino-2-((3,4-difluorophenyl)amino)thiazol-5-yl)-3-methylbutan-1-one (**37**)

Prepared following general procedure A, yellow solid, 50 mg, 46%; δ_H_ (500 MHz, DMSO-*d*_6_): 10.84 (s, 1H, NH), 7.95 (ddd, *J* = 13.2, 7.3 and 2.6 Hz, 1H, ArH), 7.41 (d, *J* = 9.1 Hz, 1H), 7.80 (bs, 2H, NH_2_), 7.26 (m, 1H, ArH), 2.23 (br d, *J* = 6.9 Hz, 2H), 2.10 (sept, *J* = 6.8 Hz, 1H), 0.91 (d, *J* = 6.8 Hz, 6H); HRMS (ES+) calcd for [C_14_H_15_F_2_N_3_OS + H], 312.0968; found, 312.0977.

##### 1-(4-Amino-2-((3,4-difluorophenyl)amino)thiazol-5-yl)-3-hydroxy-3-methylbutan-1-one (**38**)

Prepared following general procedure A with 1-bromo-4-hydroxy-4-methylpentan-2-one, 892 mg, 91%, 3 mmol scale; δ_H_ (500 MHz, MeOD): 10.87 (bs, 1H, NH), 7.95 (ddd, *J* = 13.0, 7.4 and 2.5 Hz, 1H, ArH), 7.91 (bs, 2H, NH_2_), 7.43–7.42 (m, 1H, ArH), 7.29–7.26 (m, 1H, ArH), 4.75 (bs, 1H, OH), 2.48 (s, 2H, CH_2_), 1.20 (s, 6H, 2 × CH_3_); δ_C_ (125 MHz, CDCl_3_): 189.3 (C=O), 167.5, 163.8, 117.5, 117.4, 115.5, 109.4, 109.22, 95.9 (ArC), 70.7, 51.4, 29.1; LCMS (ES+) *m*/*z*: (%) 328 [M + H]^+^*t*_R_ 4.1–4.2 (20–90% MeCN, basic); HRMS (ES+) calcd for [C_14_H_15_F_2_N_3_O_2_S + H], 328.0926; found, 328.0940.

##### 1-(4-Amino-2-((3,4-difluorophenyl)amino)thiazol-5-yl)-3-methoxy-3-methylbutan-1-one (**39**)

Prepared following general procedure A, 54 mg, 32%; δ_H_ (500 MHz, CDCl_3_): 7.56 (bs, 1H, NH), 7.45–7.40 (m, 1H, ArH), 7.15 (dd, *J* = 18.5 and 8.9 Hz, ArH), 7.05–7.01 (m, 1H, ArH), 3.22 (s, 2H, CH_2_), 1.59 (bs, 3H, CH_3_), 1.30 (s, 3H, CH_3_), 1.21 (d, *J* = 1.6 Hz, 3H, CH_3_); LCMS (ES+) *m*/*z*: (%) 342 [M + H]^+^*t*_R_ 4.60 (20–95% MeCN, acidic); HRMS (ES+) calcd for [C_15_H_17_F_2_N_3_O_2_S + H], 342.1082; found, 342.1083.

##### 1-(4-Amino-2-((3,4-difluorophenyl)amino)thiazol-5-yl)-3,3-dimethylbutan-1-one (**40**)

Prepared following general procedure A, 93 mg, 57%; δ_H_ (500 MHz, CDCl_3_): 7.45 (ddd, *J* = 11.5, 6.8 and 2.7 Hz, 1H, ArH), 7.21 (dd, *J* = 18.5 and 8.7 Hz, 1H, ArH), 7.08–7.05 (m, 1H, AH), 2.35 (s, 2H, CH_2_); LCMS (ES+) *m*/*z*: (%) 326 [M + H]^+^*t*_R_ 5.02 (20–95% MeCN, acidic).

##### 1-(4-Amino-2-((3,4-difluorophenyl)amino)thiazol-5-yl)-3-fluoro-3-methylbutan-1-one (**41**)

Prepared following general procedure A, 57 mg, 35%; δ_H_ (500 MHz, CDCl_3_): 7.49 (bs, 1H, NH), 7.44 (ddd, *J* = 11.5, 6.8 and 2.7 Hz, 1H, ArH), 7.23–7.18 (m, 1H, ArH), 7.09–7.06 (m, 1H, ArH), 2.80 (d, *J* = 17.7 Hz, 2H, CH_2_), 1.55 (s, 3H, CH_3_), 1.52 (s, 3H, CH_3_); LCMS (ES+) *m*/*z*: (%) 330 [M + H]^+^*t*_R_ 5.4–5.5 (5–95% MeCN, basic); HRMS (ES+) calcd for [C_14_H_14_F_3_N_3_OS + H], 330.0882; found, 330.0884.

##### 1-(4-Amino-2-((3,4-difluorophenyl)amino)thiazol-5-yl)-2-isopropoxyethanone (**42**)

Prepared following general procedure A, 146 mg, 45%; δ_H_ (500 MHz, DMSO-*d*_6_): 10.81 (bs, 1H, NH), 8.12–8.05 (bs, 2H, NH_2_), 8.03–7.99 (m, 1H, ArH), 7.42 (dd, *J* = 18.8 and 8.9 Hz, 1H, ArH), 7.31–7.28 (m, 1H, ArH), 3.99 (s, 2H, CH_2_), 3.68 (sep, *J* = 6.1 Hz, 1H, CH), 1.19 (d, *J* = 6.1 Hz, 6H, 2 × CH_3_). HRMS (ES+) calcd for [C_14_H_15_F_2_N_3_O_2_S + H], 328.0926; found, 328.0921.

### Experimental Details for Analogues Detailed in Table 2

#### 1-(4-Amino-2-((3,4-difluorophenyl)amino)thiazol-5-yl)-2-(*tert*-butoxy)ethanone (**43**)

Prepared following general procedure C, 7 mg, 8%; δ_H_ (500 MHz, MeOD): 7.45–7.40 (m, 1H, ArH), 7.11–7.05 (m, 1H, ArH), 7.01–6.98 (m, 1H, ArH), 3.99 (s, 2H, CH_2_), 1.21 (s, 9H, ^*t*^BuH); LCMS (ES+) *m*/*z*: (%) 342 [M + H]^+^*t*_R_ 5.3–5.4 (5–95% MeCN, basic).

#### 1-(4-Amino-2-((3,4-difluorophenyl)amino)thiazol-5-yl)-2-ethoxyethanone (**44**)

Prepared following general procedure C, 12 mg, 15%; δ_H_ (500 MHz, CDCl_3_): 7.38 (ddd, *J* = 11.6, 6.9 and 2.7 Hz, 1H, ArH), 7.09 (dd, *J* = 18.5 and 8.8 Hz, 1H, ArH), 7.01–6.97 (m, 1H, ArH), 4.04 (s, 2H, CH_2_O), 3.54 (q, *J* = 7.0 Hz, 2H, CH_2_), 1.21 (t, *J* = 7.0 Hz, 3H, CH_3_); LCMS (ES+) *m*/*z*: (%) 340 [M + H]^+^*t*_R_ 5.10–5.20 (5–95% MeCN, basic); HRMS (ES+) calcd for [C_13_H_14_F_2_N_3_O_2_S + H], 314.0769; found, 314.0771.

#### 1-(4-Amino-2-((3,4-difluorophenyl)amino)thiazol-5-yl)-3-isopropoxypropan-1-one (**45**)

Prepared following general procedure A, 60 mg, 18%; δ_H_ (500 MHz, CDCl_3_): 7.49–7.45 (m, 1H, ArH), 7.24 (bs, 1H, NH), 7.20 (dd, *J* = 18.4 and 8.7 Hz (fluorine splitting), 1H, ArH), 7.09–7.06 (m, 1H, ArH), 3.81 (t, *J* = 6.8 Hz, 2H, CH_2_), 3.64 (sep, *J* = 6.1 Hz, 1H, CH), 2.76 (t, *J* = 6.8 Hz, 2H, CH_2_), 1.18 (d, *J* = 6.1 Hz, 6H, 2 × CH_3_); LCMS (ES+) *m*/*z*: (%) 342 [M + H]^+^*t*_R_ 5.10 (5–95% MeCN, basic); HRMS (ES+) calcd for [C_15_H_17_F_2_N_3_O_2_S + H], 342.1082; found, 342.1082.

#### 1-(4-Amino-2-((3,4-difluorophenyl)amino)thiazol-5-yl)-2-cyclobutoxyethanone (**46**)

Prepared following general procedure C, 7 mg, 7%; δ_H_ (500 MHz, DMSO-*d*_6_): 7.55–7.49 (m, 1H, ArH), 7.24 (bs, 1H, NH), 7.19 (dd, *J* = 18.5 and 8.8, 1H, ArH), 7.09–7.06 (m, 1H, ArH), 4.03 (s, 4H, CH), 2.30–2.24 (m, 2H, CH), 2.09–2.01 (m, 2H, CH), 1.81–1.75 (m, 1H, CH); LCMS (ES+) *m*/*z*: (%) 340 [M + H]^+^*t*_R_ 5.40 (5–95% MeCN, basic).

#### 1-(4-Amino-2-((3,4-difluorophenyl)amino)thiazol-5-yl)-2-(2,2,3,3-tetrafluorocyclobutoxy)ethanone (**47**)

Prepared following general procedure C, 0.42 mmol, 20 mg, 12%; δ_H_ (500 MHz, CDCl_3_): 7.35 (ddd, *J* = 14.3, 6.9 and 2.7 Hz, 1H, ArH), 7.22 (bs, 1H, NH), 7.07 (dd, *J* = 18.4 and 8.8 Hz, 1H, ArH), 6.97–6.94 (m, 1H, ArH), 4.20 (d, *J* = 14.9 Hz, 1H, CH_2_), 4.06 (d, *J* = 14.9, 1H, CH_2_), 2.81–2.70 (m, 1H, CH), 2.49–2.38 (m, 1H, CH); LCMS (ES+) *m*/*z*: (%) 412 [M + H]^+^*t*_R_ 5.1 (5–95% MeCN, basic).

#### 1-(4-Amino-2-((3,4-difluorophenyl)amino)thiazol-5-yl)-2-((1,3-difluoropropan-2-yl)oxy)ethanone (**48**)

Prepared following general procedure C, 45 mg, 25%; δ_H_ (500 MHz, CDCl_3_): 7.54–7.49 (m, 1H, ArH), 7.25 (bs, 1H, NH), 7.19–7.17 (m, 1H, ArH), 7.10–7.07 (m, 1H, ArH), 4.71–4.67 (m, 2H, CH_2_F), 4.63–4.57 (m, 2H, CH_2_F), 4.35 (s, 2H, CH_2_O), 4.00–3.91 (m, 1H, CH); LCMS (ES+) *m*/*z*: (%) 364 [M + H]^+^*t*_R_ 4.8 (5–95% MeCN, basic); HRMS (ES+) calcd for [C_14_H_13_F_4_N_3_O_2_S + H], 364.0737; found, 364.0745.

#### 1-(4-Amino-2-((3,4-diflurorophenyl)amino)thiazol-5-yl)-2-(((1,1,1,3,3,3)-hexafluoropropan-2-yl)oxy)ethanone (**49**)

Prepared following general procedure C, 7 mg, 6%; δ_H_ (500 MHz, CDCl_3_): 7.57 (bs, 1H, NH), 7.49 (ddd, *J* = 11.6, 6.9 and 2.8 Hz, 1H, ArH), 7.21–7.14 (m, 1H, ArH), 7.08–7.05 (m, 1H, ArH), 4.49 (s, 2H, CH_2_), 4.32 (sept, *J* = 5.9 Hz, CH); LCMS (ES+) *m*/*z*: (%) 436 [M + H]^+^*t*_R_ 5.1–5.2 (5–95% MeCN, basic).

#### 1-(4-Amino-2-((3,4-difluorophenyl)amino)thiazol-5-yl)-2-((1,1,1-trifluoropropan-2-yl)oxy)ethanone (**50**)

Prepared following general procedure C, beige solid (80 mg, 17%); δ_H_ (500 MHz, CDCl_3_): 7.53 (m, 1H, ArH), 7.37 (bs, 1H, NH), 7.20 (m, 1H, ArH), 7.09 (m, 1H, ArH), 4.37 (d, *J* = 15.0 Hz, 1H, CH_2_), 4.27 (d, *J* = 15.0 Hz, 1H, CH_2_), 3.94 (sept, *J* = 6.6 Hz, 1H, CH), 1.46 (m, 3H); HRMS (ES+) calcd for [C_14_H_12_F_5_N_3_O_2_S + H], 382.0656; found, 382.0643.

#### 1-(4-Amino-2-((4-chlorophenyl)amino)thiazol-5-yl)-2-((1,1,1-trifluoropropan-2-yl)oxy)ethanone (**51**)

Prepared following general procedure C, 15 mg, 14%; δ_H_ (500 MHz, CDCl_3_): 7.45 (s, 4H, ArH), 4.44 (d, *J* = 14.8 Hz, 1H, CH_2_), 4.33 (d, *J* = 14.8 Hz, 1H, CH_2_), 4.01 (sep, *J* = 6.4 Hz, 1H, CH), 1.51 (dd, *J* = 6.4 and 0.4 Hz, 3H, CH_3_); LCMS (ES+) *m*/*z*: (%) 380 [M + H]^+^*t*_R_ 4.3–4.5 (5–95% MeCN, basic).

#### 1-(4-Amino-((4-(trifluoromethyl)phenyl)amino)thiazol-5-yl)-2-((1,1,1-trifluoropropan-2-yl)oxy)ethanone (**52**)

Prepared following general procedure C, beige solid, 34 mg, 28%; δ_H_ (500 MHz, DMSO-*d*_6_): 11.07 (s, 1H, NH), 8.08 (bs, 2H, NH_2_), 7.87 (d, *J* = 8.6 Hz, 2H, ArH), 7.69 (d, *J* = 8.6 Hz, 2H, ArH), 4.24–4.31 (m, 3H, CH, and CH_2_), 1.36 (d, *J* = 6.5 Hz, 3H, CH_3_); LCMS (ES+) *m*/*z*: (%) 414 [M + H]^+^*t*_R_ 5.2 (5–95% MeCN, basic); HRMS (ES+) calcd for [C_15_H_13_F_6_N_3_O_2_S + H], 414.0705; found, 414.0707.

#### 1-(4-Amino-2-((2,2-difluorobenzo[*d*][1,3]dioxol-5-yl)amino)thiazol-5-yl)-2-((1,1,1-trifluoropropan-2-yl)oxy)ethan-1-one (**53**)

Prepared following general procedure C, 7 mg, 21%; δ_H_ (500 MHz, CDCl_3_): 7.57 (bs, 1H, NH), 7.43 (d, *J* = 2.0 Hz, 1H, ArH), 7.08 (d, *J* = 8.6 Hz, 1H, ArH), 7.03 (dd, *J* = 8.6 and 2.0 Hz, 1H, ArH), 4.37 (d, *J* = 14.8 Hz, 1H, CH_2_), 4.27 (d, *J* = 14.8 Hz, 1H, CH_2_), 3.94 (sep, *J* = 6.5 Hz, 1H, CH), 1.45 (d, *J* = 6.5 Hz, 3H, CH_3_); LCMS (ES+) *m*/*z*: (%) 426 [M + H]^+^*t*_R_ 4.55 (20–95% MeCN, acidic).

#### 1-(4-Amino-2-((3-(difluoromethoxy)phenyl)amino)thiazol-5-yl)-2-((1,1,1-trifluoropropan-2-yl)oxy)ethanone (**54**)

Prepared following general procedure B and then C, 10 mg, 12%; δ_H_ (500 MHz, CDCl_3_): 7.21 (t, *J* = 8.2 Hz, 1H, ArH), 7.16 (t, *J* = 2.1 Hz, 1H, ArH), 7.02 (dd, *J* = 8.1 and 2.1 Hz, 1H, ArH), 6.76 (d, *J* = 8.1 Hz, 1H, ArH), 6.44 (t, *J* = 73.4 (Fluorine split), 1H, OCHF_2_), 4.20 (d, *J* = 14.3 Hz, 1H, CH_2_), 4.09 (d, *J* = 14.3 Hz, 1H, CH_2_), 3.75 (sep, *J* = 6.4 Hz, 1H, CH), 1.27 (d, *J* = 6.5 Hz, 3H, CH_3_); LCMS (ES+) *m*/*z*: (%) 412 [M + H]^+^*t*_R_ 4.40 (20–95% MeCN, basic).

#### 1-(4-Amino-2-((1-methyl-1*H*-pyrazol-3-yl)amino)thiazol-5-yl)-2-((1,1,1-trifluoropropan-2-yl)oxy)ethan-1-one (**55**)

Prepared following general procedure A, beige solid, 43 mg, 13%; δ_H_ (500 MHz, DMSO-*d*_6_): 11.18 (s, 1H, NH), 7.89 (bs, 2H, NH_2_), 7.63 (d, *J* = 2.6 Hz, 1H, ArH), 6.03 (s, 1H, ArH), 4.24–4.31 (m, 3H, CH, and CH_2_), 3.77 (s, 3H, CH_3_), 1.38 (d, *J* = 6.5 Hz, 3H, CH_3_); LCMS (ES+) *m*/*z*: (%) 350 [M + H]^+^*t*_R_ 4.50 (5–95% MeCN, basic); HRMS (ES+) calcd for [C_12_H_15_F_3_N_5_O_2_S + H], 350.0893; found, 350.0900.

#### 1-(4-Amino-2-((6-(trifluoromethyl)pyridine-3-yl)amino)thiazol-5-yl)-2-((1,1,1-trifluoropropan-2-yl)oxy)ethanone (**56**)

Prepared following general procedure D, 72 mg, 52%; δ_H_ (500 MHz, CDCl_3_): 8.62 (d, *J* = 2.7 Hz, py-H), 8.23 (dd, *J* = 8.7 and 2.7 Hz, 1H, py-H), 7.61 (d, *J* = 8.7 Hz, 1H, py-H), 7.48 (bs, 1H, NH), 4.27 (d, *J* = 14.8 Hz, 1H, CH_2_), 4.18 (d, *J* = 14.8 Hz, 1H, CH_2_), 3.84 (sep, *J* = 6.5 Hz, 1H, CH), 1.36 (dd, *J* = 6.5 and 0.6 Hz, CH_3_); LCMS (ES+) *m*/*z*: (%) 415 [M + H]^+^*t*_R_ 4.7–4.8 (5–95% MeCN, basic); HRMS (ES+) calcd for [C_22_H_11_FN_4_O_2_S + H], 415.0658; found, 415.0673.

#### 1-(4-Amino-2-((6-methoxypyridin-3-yl)amino)thiazol-5-yl)-2-((1,1,1-trifluoropropan-2-yl)oxy)ethanone (**57**)

Prepared following general procedure A, reddish solid, 45 mg, 22%; δ_H_ (500 MHz, DMSO-*d*_6_): 10.66 (s, 1H, NH), 8.44 (d, *J* = 2.7 Hz, 1H, ArH), 8.05 (bs, 2H, NH_2_), 7.96 (dd, *J* = 8.9 and 2.7 Hz, 1H, ArH), 6.85 (d, *J* = 8.9 Hz, 1H, ArH), 4.27 (sept, *J* = 6.6 Hz, 1H, CH), 4.24 (d, *J* = 14.9 Hz, 1H, CH_2_), 4.21 (d, *J* = 14.9 Hz, 1H, CH_2_), 3.83 (s, 3H, OCH_3_), 1.33 (d, *J* = 6.6 Hz, 3H, CH_3_); LCMS (ES+) *m*/*z*: (%) 377 [M + H]^+^*t*_R_ 4.8 (5–95% MeCN, basic); HRMS (ES+) calcd for [C_14_H_15_F_3_N_4_O_3_S + H], 377.0890; found, 377.0897.

#### 1-(4-Amino-2-((3,4-difluorophenyl)amino)thiazol-5-yl)-2-(pyrrolidin-1-yl)ethanone (**58**)

Prepared following general procedure E, beige solid, 46 mg, 59%; δ_H_ (500 MHz, DMSO-*d*_6_): 10.69 (s, 1H, NH), 8.01 (m, 1H, ArH), 7.96 (bs, 2H, NH_2_), 7.39 (q, *J* = 9.6 Hz, 1H, ArH), 7.28 (m, 1H, ArH), 3.22 (s, 2H, CH_2_), 2.55 (m, 4H, 2 × CH_2_), 1.76 (m, 4H, 2 × CH_2_); LCMS (ES+) *m*/*z*: (%) 339 [M + H]^+^*t*_R_ 4.33; HRMS (ES+) calcd for [C_15_H_16_F_2_N_4_OS + H], 339.1086; found, 339.1091.

#### 1-(4-Amino-2-((3,4-difluorophenyl)amino)thiazol-5-yl)-2-(2-methylpyrrolidin-1-yl)ethanone (**59**)

Prepared following general procedure E, brownish solid, 40 mg, 37%; δ_H_ (500 MHz, DMSO-*d*_6_): 10.69 (s, 1H, NH), 8.01 (m, 1H, ArH), 7.96 (bs, 2H, NH_2_), 7.39 (q, *J* = 9.5 Hz, 1H, ArH), 7.28 (m, 1H, ArH), 3.52 (d, *J* = 15.5 Hz, 1H, CH_2_), 2.96 (m, 1H, CH), 2.81 (d, *J* = 15.5 Hz, 1H, CH_2_), 2.17 (m, 1H, CH_2_), 1.96 (m, 1H, CH_2_), 1.73 (m, 1H, CH_2_), 1.70 (m, 1H, CH_2_), 1.40 (m, 1H, CH_2_), 1.10 (d, *J* = 5.9 Hz, 3H, CH_3_); LCMS (ES+) *m*/*z*: (%) 353 [M + H]^+^*t*_R_ 5.2–5.6 (5–95% MeCN, basic); HRMS (ES+) calcd for [C_16_H_18_F_2_N_4_OS + H], 353.1242; found, 353.1243.

#### 1-(4-Amino-2-((3,4-difluorophenyl)amino)thiazol-5-yl)-2-(2-(trifluoromethyl)pyrrolidin-1-yl)ethanone (**60**)

Prepared following general procedure E, brownish solid 25 mg, 26%; δ_H_ (500 MHz, DMSO-*d*_6_): 10.79 (s, 1H, NH), 7.98 (ddd, *J* = 13.3, 7.4 and 2.7 Hz, 1H, ArH), 7.99 (bs, 2H, NH_2_), 7.40 (q, *J* = 9.1 Hz, 1H, ArH), 7.29 (m, 1H, ArH), 3.69 (d, *J* = 15.9 Hz, 1H, CH_2_), 3.54 (m, 1H, CH_2_), 3.32 (d, *J* = 15.9 Hz, 1H, CH_2_), 3.02 (t, *J* = 7.5 Hz, 1H, CH), 2.47 (m, 1H, CH_2_), 1.84 (m, 2H, CH_2_), 2.11 (m, 1H, CH_2_), 1.76 (m, 1H, CH_2_); LCMS (ES+) *m*/*z*: (%) 407 [M + H]^+^*t*_R_ 5.28 (20–95% MeCN, acidic); HRMS (ES+) calcd for [C_16_H_15_F_5_N_4_OS + H], 407.0959; found, 407.0976.

#### 1-(4-Amino-2-((3,4-difluorophenyl)amino)thiazol-5-yl)-2-(piperidin-1-yl)ethanone (**61**)

Prepared following general procedure E, brownish solid, 90 mg, 16%; δ_H_ (500 MHz, DMSO-*d*_6_): 10.70 (s, 1H, NH), 8.01 (ddd, *J* = 13.3, 7.4 and 2.5 Hz, 1H, ArH), 7.94 (b, 2H, NH_2_), 7.40 (q, *J* = 9.1 Hz, 1H, ArH), 7.29 (m, 1H, ArH), 3.06 (s, 2H, CH_2_), 2.38 (m, 4H, 2 × CH_2_), 1.59 (m, 4H, 2 × CH_2_), 1.41 (m, 2H, CH_2_); LCMS (ES+) *m*/*z*: (%) 353 [M + H]^+^*t*_R_ 5.4–5.6 (5–95% MeCN, basic); HRMS (ES+) calcd for [C_16_H_18_F_2_N_4_OS + H], 353.1242; found, 353.1243.

#### 1-(4-Amino-2-((3,4-difluorophenyl)amino)thiazol-5-yl)-2-morpholinoethanone (**62**)

Prepared following general procedure E, yellowish solid, 72 mg, 40%; δ_H_ (500 MHz, DMSO-*d*_6_): 10.75 (s, 1H, NH), 8.01 (ddd, *J* = 13.3, 7.4 and 2.5 Hz, 1H, ArH), 7.97 (bs, 2H, NH_2_), 7.40 (q, *J* = 9.1 Hz, 1H, ArH), 7.29 (m, 1H, ArH), 3.66 (m, 4H, 2 × CH_2_), 3.07 (s, 2H, CH_2_), 2.44 (m, 4H, 2 × CH_2_); LCMS (ES+) *m*/*z*: (%) 355 [M + H]^+^*t*_R_ 4.8–5.0 (5–95% MeCN, basic); HRMS (ES+) calcd for [C_15_H_16_F_2_N_4_O_2_S + H], 355.1035; found, 355.1043.

#### 1-(4-Amino-2-((3,4-difluorophenyl)amino)thiazol-5-yl)-2-((2*S*,6*R*)-2,6-dimethylmorpholino)ethanone (**63**)

Prepared following general procedure E, yellowish solid 127 mg, 33%; δ_H_ (500 MHz, DMSO-*d*_6_): 10.72 (s, 1H, NH), 8.00 (ddd, *J* = 13.3, 7.4 and 2.5 Hz, 1H, ArH), 7.97 (bs, 2H, NH_2_), 7.40 (q, *J* = 9.1 Hz, 1H, ArH), 7.28 (m, 1H, ArH), 3.74 (m, 2H, 2 × CH), 3.05 (s, 2H, CH_2_), 2.69 (d, *J* = 10.8 Hz, 2H, 2 × CH_2_), 1.78 (t, *J* = 10.8 Hz, 2H, 2 × CH_2_), 1.06 (d, *J* = 6.3 Hz, 6H, 2 × CH_3_); LCMS (ES+) *m*/*z*: (%) 383 [M + H]^+^*t*_R_ 4.51 (5–90% MeCN, acidic); HRMS (ES+) calcd for [C_17_H_20_F_2_N_4_O_2_S + H], 383.1348; found, 383.1365.

#### 1-(4-Amino-2-((3,4-difluorophenyl)amino)thiazol-5-yl)-3-morpholinopropan-1-one (**64**)

Prepared following general procedure E, colorless solid 148 mg, 25%; δ_H_ (500 MHz, DMSO-*d*_6_): 10.87 (s, 1H, NH), 7.96 (ddd, *J* = 13.1, 7.3 and 2.5 Hz, 1H, ArH), 7.78 (bs, 2H, NH_2_), 7.41 (q, *J* = 9.1 Hz, 1H, ArH), 7.26 (m, 1H, ArH), 3.55 (t, *J* = 4.5 Hz, 4H, 2 × CH_2_), 2.60 (m, 2H, CH_2_), 2.52 (m, 2H, CH_2_), 2.37 (m, 4H, 2 × CH_2_); LCMS (ES+) *m*/*z*: (%) 369 [M + H]^+^*t*_R_ 4.37 (5–90% MeCN, acidic); HRMS (ES+) calcd for [C_16_H_18_F_2_N_4_O_2_S + H], 369.1191; found, 369.1190.

#### 1-(4-Amino-2-((3,4-difluorophenyl)amino)thiazol-5-yl)-2-((1*S*,4*S*)-2-oxa-5-azabicyclo[2.2.1]heptan-5-yl)ethanone (**65**)

Prepared following general procedure E, yellowish solid, 333 mg, 52%; δ_H_ (500 MHz, DMSO-*d*_6_): 10.70 (s, 1H, NH), 8.01 (ddd, *J* = 13.3, 7.4 and 2.5 Hz, 1H, ArH), 7.96 (bs, 2H, NH_2_), 7.40 (q, *J* = 9.1 Hz, 1H, ArH), 7.28 (m, 1H, ArH), 4.39 (bs, 1H, CH), 3.92 (d, *J* = 7.2 Hz, 1H, CH), 3.53 (m, 2H, CH_2_), 3.36 (d, *J* = 16.0 Hz, 1H, CH_2_), 3.28 (d, *J* = 16.0 Hz, 1H, CH_2_), 2.81 (d, *J* = 9.5 Hz, 1H, CH_2_), 2.45 (d, *J* = 9.5 Hz, 1H, CH_2_), 1.83 (bd, *J* = 9.6 Hz, 1H, CH_2_), 1.64 (bd, *J* = 9.6 Hz, 1H, CH_2_); LCMS (ES+) *m*/*z*: (%) 367 [M + H]^+^*t*_R_ 3.71 (5–90% MeCN, acidic); HRMS (ES+) calcd for [C_16_H_16_F_2_N_4_O_2_S + H], 367.1035; found, 367.1048.

#### 1-(4-Amino-2-((3,4-difluorophenyl)amino)thiazol-5-yl)-2-((1*R*,5*S*)-8-oxa-3-azabicyclo[3.2.1]octan-3-yl)ethanone (**66**)

Prepared following general procedure E, yellowish solid 84 mg, 22%; δ_H_ (500 MHz, DMSO-*d*_6_): 10.78 (s, 1H, NH), 7.99 (ddd, *J* = 13.3, 7.4 and 2.5 Hz, 1H, ArH), 7.89 (bs, 2H, NH_2_), 7.41 (q, *J* = 9.2 Hz, 1H, ArH), 7.29 (m, 1H, ArH), 4.23 (bs, 2H, 2 × CH), 3.00 (s, 2H, CH_2_), 2.55 (d, *J* = 11.1 Hz, 2H, 2 × CH_2_), 2.23 (bd, *J* = 11.1 Hz, 2H, 2 × CH_2_), 2.09 (m, 2H, CH_2_), 1.78 (m, 2H, CH_2_); LCMS (ES+) *m*/*z*: (%) 381 [M + H]^+^*t*_R_ 4.40 (5–90% MeCN, acidic); HRMS (ES+) calcd for [C_17_H_18_F_2_N_4_O_2_S + H], 381.1191; found, 381.1210.

#### 1-(4-Amino-2-((3,4-difluorophenyl)amino)thiazol-5-yl)-2-(3,3-difluoropiperidin-1-yl)ethanone (**67**)

Prepared following general procedure E, yellowish solid, 26 mg, 22%; δ_H_ (500 MHz, DMSO-*d*_6_): 10.78 (s, 1H, NH), 7.99 (m, 1H, ArH), 7.96 (bs, 2H, NH_2_), 7.40 (q, *J* = 9.5 Hz, 1H, ArH), 7.29 (m, 1H, ArH), 3.18 (s, 2H, CH_2_), 2.77 (t, *J* = 11.9 Hz, 2H, CH_2_), 2.42 (m, 2H, CH_2_), 1.91 (m, 2H, CH_2_), 1.73 (m, 2H, CH_2_); LCMS (ES+) *m*/*z*: (%) 383 [M + H]^+^*t*_R_ 4.51 (5–95% MeCN, basic); HRMS (ES+) calcd for [C_16_H_16_F_4_N_4_OS + H], 389.1054; found, 389.1059.

#### 1-(4-Amino-2-((3,4-difluorophenyl)amino)thiazol-5-yl)-2-(4,4-difluoropiperidin-1-yl)ethanone (**68**)

Prepared following general procedure E, brownish solid, 37 mg, 31%; δ_H_ (500 MHz, DMSO-*d*_6_): 10.73 (s, 1H, NH), 8.02 (m, 1H, ArH), 7.96 (bs, 2H, NH_2_), 7.40 (q, *J* = 9.1 Hz, 1H, ArH), 7.28 (m, 1H, ArH), 3.16 (s, 2H, CH_2_), 2.59 (m, 4H, 2 × CH_2_), 2.05 (m, 4H, 2 × CH_2_); LCMS (ES+) *m*/*z*: (%) 389 [M + H]^+^*t*_R_ 5.0–5.3 (5–95% MeCN, basic); HRMS (ES+) calcd for [C_16_H_16_F_4_N_4_OS + H], 389.1054; found, 389.1058.

## Compound Preparation

The preparation of individual compounds is described in the Supporting Information.

### Cell-Based Assays

Drug sensitivity assays with BSF *T. b. brucei* “single marker” S427 and human MRC-5 fibroblasts were conducted as previously described.^43^

#### Static–Cidal Assay

An assay to assess initial indications of the cidal nature of the compound series was conducted as previously reported.^15^ In brief, BSF trypanosomes were seeded into 384-well plates at 4 × 10^5^ cells mL^–1^ (50 μL/well), followed by immediate addition of resazurin (50 μM final) to one of the plates, and all plates were incubated at 37 °C and 5% CO_2_. After 4 h, the time = 0 plate was read using a PerkinElmer Victor 3 plate reader (excitation 528 nm; emission 590 nm). Twenty hours later, the second plate was processed in the same way, and at 44 h, the last plate was processed. The minimum cidal concentration was defined as the lowest concentration of the drug that resulted in a decrease of resorufin signal over time. For dose–response curves from this assay, either a monophasic or a biphasic equation was used depending on which one provided the best fit. For monophasic fits, the following 4-parameter equation was used
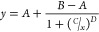
where *A* = % inhibition at the bottom, *B* = % inhibition at the top, *C* = EC_50_, *D* = slope, *x* = inhibitor concentration, and *y* = % inhibition. For biphasic fits, the following equation
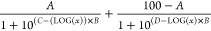
was used, with *A* = % inhibition at the midplateau, *B* = slope, *C* = log [SC EC_50_ (1)], and *D* = log [SC EC_50_(2)]. Thus, SC EC_50_ (1) is the EC_50_ for the first phase of the curve, and SC EC_50_ (2) is the EC_50_ for the second phase of the curve. Inhibition at the bottom of the curve is fixed at 0% and at the top at 100%.

### Drug Metabolism and Pharmacokinetics

#### In Vivo Studies

Test compounds (**16**, **38**) were dosed via IP injection of 10 mg kg^–1^ free base (**16**, **38**), dose volume; 10 mL kg^–1^; dose vehicle, 5% DMSO/40% PEG400/55% Milli-Q (**16**), and 15% solutol/85% Milli-Q (**38**) (*n* = 3) to female NMRI (**16**, **38**) or HRN mice (**16**). To determine compound exposure, compounds were dosed by gavage (**16**, **38**) at 10 mg kg^–1^ free base (**16**, **38**), dose volume; and 5 mL kg^–1^ (**16**, **38**), dose vehicle (10% DMSO/40% PEG400/50% Milli-Q) to female NMRI mice, *n* = 3/dose level (**16**), or Sprague Dawley rats (**38**). Blood samples were taken from each mouse/rat at 5, 15, and 30 min, 1, 2, 4, 6, and 8 h postdose and mixed with two volumes of distilled water. After suitable sample preparation, the concentration of the test compound in blood was determined by UPLC–MS/MS using a Quattro Premier XE (Waters, USA). Pharmacokinetic parameters were derived from the mean blood concentration–time curve using PKsolutions software v 2.0 (Summit Research Services, USA).

### Mouse Brain Penetration

Each compound was dosed as an IV bolus at 10 mg kg^–1^ dissolved in 15% solutol/Milli-Q (dose volume 10 mL kg^–1^) to female NMRI mice (*n* = 3). At 30 min following the IV bolus of the test compound, mice (*n* = 3/time point) were placed under terminal anesthesia with isoflurane. A blood sample was taken by cardiac puncture and added to two volumes of distilled water, and the brain was removed. After suitable sample preparation, the concentration of the test compound in blood and the brain was determined by UPLC–MS/MS using a Quattro Premier XE (Waters, USA). For each mouse at each time point, the concentration in the brain (ng g^–1^) was divided by the concentration in blood (ng mL^–1^) to give a brain: blood ratio.

### Efficacy Studies

Stage 1 efficacy experiments using *T. b. brucei* S427 were performed as described,^44^ with minor modifications. In brief, male SD rats (**38**) or female HRN (**16**) and NMRI (**16**, **38**) mice (3–5 per group) were injected intraperitoneally with 1 × 10^4^ BSFs of *T. brucei*. These BSFs come from a stock of cryopreserved stabilates containing 10% glycerol. The stabilate was suspended in 20 mM Hanks’ balanced salt solution with glucose to obtain a trypanosome concentration of 5 × 10^4^ cells mL^–1^. Each mouse was injected with 0.2 mL. Compounds (**16**, **38**) were administered twice daily IP (**16**, **38**) or PO (**38**) from day 3 to day 6 (**16**) or day 3 only (**38**) of the experiment, and parasitemia levels were monitored up to day 30. Dose concentrations were 1.25, 2.5, 5, 7.5, 10, 30, and 50 mg kg^–1^ free base (**16**) or 100 mg kg^–1^ free base (**38**). The doses were prepared fresh daily, using 5% DMSO/40% PEG400/55% Milli-Q (**16**) or 15% solutol/85% Milli-Q (**38**), and the dose volume was 10 mL kg^–1^. One group of three mice was an untreated control group.

### Intrinsic Clearance Studies

Test compounds (0.5 μM) were incubated with female CD1 mouse liver microsomes (Xenotech LLC; 0.5 mg mL^–1^ 50 mM potassium phosphate buffer, pH 7.4), and the reaction started with the addition of excess NADPH (8 mg mL^–1^ 50 mM potassium phosphate buffer, pH 7.4). Immediately, at 0 min, and then at 3, 6, 9, 15, and 30 min, an aliquot (50 μL) of the incubation mixture was removed and mixed with acetonitrile (100 μL) to stop the reaction. The internal standard was added to all samples, the samples were centrifuged to sediment the precipitated protein, and the plates were then sealed prior to UPLC–MS/MS analysis using a Quattro Premier XE (Waters Corporation, USA). XLfit (IDBS, UK) was used to calculate the exponential decay and consequently the rate constant (*k*) from the ratio of the peak area of the test compound to the internal standard at each time point. The rate of intrinsic clearance (CL_int_) of each test compound was then calculated using the following calculation
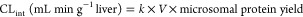
where *V* (mL mg^–1^ protein) is the incubation volume mg^–1^ protein added and the microsomal protein yield is taken as 52.5 mg protein g^–1^ liver. Verapamil (0.5 μM) was used as a positive control to confirm acceptable assay performance.

### Equilibrium Dialysis

In brief, a 96-well equilibrium dialysis apparatus was used to determine the free fraction in plasma and the brain (HT Dialysis LLC, Gales Ferry, CT). Isotonic buffer was prepared using 8.69 g of Na_2_HPO_4_, 1.9 g of KH_2_PO_4_, and 4.11 g of NaCl dissolved in 1 L of Milli-Q water, and the pH was adjusted to 7.4. Artificial CSF was prepared using 3.652 g of NaCl, 93.2 mg of KCl, 119.96 mg of MgCl_2_, 92.61 mg of CaCl_2_, and 268 mg of Na_2_HPO_4_ heptahydrate dissolved in 0.5 L of water, and the pH was adjusted to 7.4. Membranes [12–14 (plasma) or 6–9 (brain) kDA cutoff] were conditioned in deionized water for 60 min, followed by conditioning in 80:20 deionized water/ethanol for 20 min, and then rinsed in isotonic buffer (plasma) or artificial CNS (brain) before use. Female CD1 mouse plasma was defrosted and centrifuged for 10 min at 3000 rpm (Allegra X12-R, Beckman Coulter, USA). The control mouse brain was homogenized in two volumes of artificial CSF in a Covaris S2 acoustic homogenizer. Plasma or brain homogenate was spiked with the test compound (10 μg mL^–1^), and 150 μL aliquots (*n* = 3 replicate determinations) were loaded into the 96-well equilibrium dialysis plate. Dialysis versus 150 μL of isotonic buffer (plasma) or artificial CSF (brain) was carried out for 5 h in a temperature-controlled incubator at 37 °C (Barworld Scientific Ltd, UK) using an orbital microplate shaker at 125 rpm (Barworld Scientific Ltd, UK). At the end of the incubation period, aliquots of plasma/homogenized brain or buffer were transferred to a clean 96-well plate, and the composition in each well was balanced with control fluid, such that the volume of buffer to the matrix was the same. Sample extraction was performed by the addition of 400 μL of acetonitrile containing an appropriate internal standard. Samples were allowed to mix for 1 min and then centrifuged at 3000 rpm in 96-well blocks for 10 min (Allegra X12-R, Beckman Coulter, USA). All samples were analyzed by means of UPLC/MS/MS on a Quattro Premier XE or Micro TQs mass spectrometer (Waters Corporation, USA). The unbound fraction was determined as the ratio of the peak area in the buffer to that in the matrix.

### Solubility

The kinetic aqueous solubility of the test compounds was measured using laser nephelometry. Compounds were subject to serial dilution from 10 to 0.5 mM in DMSO. An aliquot was then mixed with Milli-Q water to obtain an aqueous dilution plate with a final concentration range of 13–250 μM, with a final DMSO concentration of 2.5%. Triplicate aliquots were transferred to a flat-bottomed polystyrene plate which was immediately read on the NEPHELOstar (BMG Lab Technologies). The amount of laser scatter caused by insoluble particulates (relative nephelometry units, RNUs) was plotted against compound concentration using a segmental regression fit, with the point of inflection being quoted as the compound’s aqueous solubility (μM).

### Target Deconvolution Studies

#### Compounds

GW8510 was purchased from Insight Biotechnology.

#### In Vitro Drug Sensitivity Assays

Drug sensitivity assays were carried out with BSF *T. b. brucei* “single marker” S427 or BSF *T. b. brucei*, Lister 427, MiTat 1.2, clone 221a 2T1 cells,^45^ cells grown at 37 °C with 5% CO_2_ in an HMI-9T medium^46^ as previously described.^47^ 2T1 cells were initially maintained with 1 μg mL^–1^ puromycin and 1 μg mL^–1^ phleomycin prior to transfection or 2.5 μg mL^–1^ hygromycin and 1 μg mL^–1^ phleomycin after transfection. Induction of overexpression was achieved by the addition of 1 μg mL^–1^ tetracycline to the culture medium.

### Screening and Analysis of Overexpression and RNAi Libraries

The *T. b. brucei* overexpression library was performed as described previously.^27^ The library was maintained at or above 2 × 10^7^ cells to maintain complexity in a medium containing phleomycin (1 μg mL^–1^) and blasticidin (1 μg mL^–1^). Overexpression was induced with tetracycline (1 μg mL^–1^) for 24 h and 2 × 10^7^ cells in 150 mL of media were used to initiate each screen. The library was screened with either 600 nM compound **38** or 300 nM compound **69**; the concentration of **38** was increased to 1200 nM on day 3. Cells were passaged as required, and genomic DNA was extracted after 8–9 days using a Qiagen DNeasy Blood and Tissue Kit. Overexpressed fragments were amplified using the OeseqA primer (CGGCGTACACCCTATCAATGA) in a “long-range” PCR using LongAmp polymerase and purified using a QIAquick PCR Purification Kit. The products were sequenced using an Illumina HiSeq platform at the Beijing Genomics Institute. Reads were aligned to the *T. brucei* 927 reference genome (v39.0, tritrypdb.org) with Bowtie 2 software^48^ using the conditions, very-sensitive-local. The subsequent alignment files were manipulated with SAMtools^49^ and a custom script to identify reads with barcodes (−Ff GATAGAGTGGTACCGGCCGG, −Fr CCGGCCGGTACCACTCTATC, −Rf CAATGATAGAGTGGCCGGCC, and −Rr GGCCGGCCACTCTATCATTG), which also revealed insert orientation.^27^ Total and barcoded reads were then quantified using the Artemis genome browser^50^ and Excel.

RIT-seq library screens were performed as described previously.^21,51^ The RNAi library was maintained in the presence of blasticidin (1 μg mL^–1^) and phleomycin (1 μg mL^–1^) in the culture medium and with a minimum of 2 × 10^7^ cells. Following tetracycline (1 μg mL^–1^) induction for 24 h, compound **38** (600 nM, increased to 1200 nM on day 3) and compound **69** (600 nM) were added to cultures and supplemented with fresh compounds and tetracycline as required. DNA was extracted from compound-selected cells, and RNAi target fragments were amplified from compound-selected parasites by PCR using the Lib2f and Lib2r primers.^51^ PCR products were fragmented and sequenced with an Illumina HiSeq platform at Beijing Genomics Institute (BGI). Reads were mapped to the *T. brucei* 927 reference genome (v39; tritrypdb.org) using Bowtie2 software^48^ with the following parameter: very-sensitive-local. Following manipulation with SAMtools,^49^ the alignment files were searched with a custom script to identify reads with the following barcode: GCCTCGCGA.^51^ The total and barcoded reads were then quantified using the Artemis genome browser.^50^

### Generation of Drug-Resistant Parasites and Whole Genome Sequencing

Compound-resistant cell lines were generated by subculturing a clone of *T. brucei* in the continuous presence of **38**. Starting at sublethal concentrations, drug concentrations in two independent cultures were increased in a stepwise manner, usually 2-fold. When parasites were able to survive and grow in concentrations of drugs equivalent to more than 10 times the established EC_50_ value, the resulting cell lines were cloned by limiting dilution in the presence of the compound. Two clones (RES I-II) were selected for further biological study. A standard alkaline lysis protocol was used to isolate genomic DNA from compound-resistant bloodstream *T. brucei* parasites (∼1 × 10^8^). Whole genome sequencing was performed using a DNB-seq next-generation sequencing platform (BGI, Hong Kong). Sequencing reads (150 bp) were aligned to the *T. brucei* TREU927 genome (v39; tritrypDB) using Bowtie2^48^ and Samtools^49^ software. At least 50-fold genome coverage was achieved for all samples. Samtools and BCFtools^52^ (mpileup) were used to call SNP and indels compared with the wild-type starter clone, where the overall quality score (QUAL) was >100. Artemis^50^ was used to analyze chromosome and gene copy number variation, as well as visualization of SNPs.

### Generation and Transfection of Overexpression Vectors

The following primers were used to PCR amplify Tb927.8.6290 from genomic DNA isolated from wild-type and **38**-resistant trypanosomes 5′-CGCG**TTAATTAA**ATGGAAGACGCGGTAGAGGC-3′ (PacI site in bold) and 5′-GCGC**GGATCC**TTAGCAATCTTTTGAAACAACACTTGAC-3′ (BamHI site in bold). The PCR products (1272 bp) were then cloned into the pRPa plasmid.^53^ The accuracy of the plasmid constructs was confirmed by in-house Sanger sequencing and then linearized with AscI prior to transfection. The linearized plasmids were introduced into 2T1 *T. b. brucei* cells following removal of puromycin from the media and selected with 2.5 μg mL^–1^ hygromycin and 1 μg mL^–1^ phleomycin. Two independent clones were selected for further studies.

### Proteomic Analysis of Overexpression—Sample Preparation

Overexpression of wild-type and mutated versions of the hypothetical protein encoded by Tb927.8.6290 was achieved by the addition of tetracycline to the culture medium for 24 h. Following induction, samples (2 × 10^7^ cells) were washed once with 1× PBS and then lysed with 30 μL of lysis buffer (1× PBS with 2× Roche protease inhibitor and 1% NP40). Samples were centrifuged at 15,000*g* for 10 min at 4 °C, and the supernatant was transferred to a new tube with 10 μL of SDS page buffer and 2 μL of DDT (50 mM final). Samples were boiled for 5 min at 95 °C and then run on a NuPAge gel for 8 min. Samples were run 1.5 cm into a bis–Tris 10% (w/v) acrylamide gel and stained with Coomassie quick reagent for 30 min. The entire gel bands were removed and subjected to in-gel reduction with 10 mM dithiothreitol, alkylation with 50 mM iodoacetamide, and digestion with 12.5 μg mL^–1^ trypsin (Pierce) for >16 h at 37 °C. Recovered tryptic peptides were then vacuum-dried prior to analysis.

### Confirmation of Target Overexpression—LC–MS/MS Analysis

Analysis of the peptide readout was performed on a Q Exactive Plus, mass spectrometer (Thermo Scientific) coupled with a Dionex Ultimate 3000 RS (Thermo Scientific). LC buffers used were the following: buffer A [0.1% formic acid in Milli-Q water (v/v)] and buffer B [80% acetonitrile and 0.1% formic acid in Milli-Q water (v/v)]. Aliquots of 15 μL per sample were loaded at 10μL/min onto a trap column (100 μm × 2 cm, PepMap nanoViper C18 column, 5 μm, 100 Å, Thermo Scientific) which was equilibrated with 98% Buffer A. The trap column was washed for 5 min at the same flow rate, and then the trap column was switched in line with a Thermo Scientific, resolving the C18 column (75 μm × 50 cm, PepMap RSLC C18 column, 2 μm, 100 Å). The peptides were eluted from the column at a constant flow rate of 300 nL/min with a linear gradient from 2% buffer to 35% buffer B in 125 min and then to 98% buffer B in 127 min. The column was then washed with 98% buffer B for 20 min and re-equilibrated in 2% buffer B for 17 min. Q Exactive Plus was used in data-dependent mode. A scan cycle involved an MS1 scan (*m*/*z* range from 335 to 1600), with a maximum ion injection time of 20 ms, a resolution of 70,000, and an automatic gain control (AGC) value of 1 × 10^6^ followed by 15 sequential dependent MS2 scans (with an isolation window set to 1.4 Da, resolution at 17,500, maximum ion injection time at 100 ms, and AGC 2 × 10^5^. The stepped collision energy was set to 27 and fixed first mass to 100 *m*/*z*. Spectrum was acquired in centroid mode and unassigned charge states, charge states above 6, as well as singly charged species were rejected. To ensure mass accuracy, the mass spectrometer was calibrated on the first day that the runs were performed. LC–MS analysis was performed by the FingerPrints Proteomics Facility (University of Dundee).

### Proteomics Data Analysis

MS data analysis was performed using the software MaxQuant (http://maxquant.org, version 2.0.3.0). Carbamidomethyl (C), oxidation (M), acetyl (protein N-term), deamidation (NQ), and Gln- > pyro-Glu were set as variable modifications. Proteins were identified by searching a protein sequence database containing *T. brucei* TREU927 annotated proteins (downloaded from TriTrypDB 50, http://www.tritrypdb.org). LFQ and “March between runs” features were enabled. Trypsin/P and Lysc/P were selected as the digestive enzymes with two potential missed cleavages. The FDR threshold for peptides and proteins was 0.01. The FTMS MS/MS mass tolerance was set to 10 ppm, and the ITMS MS/MS mass tolerance was 0.6 Da. Protein abundance was obtained from LFQ intensity values. LFQ intensities were calculated using at least two unique peptides. Data was visualized using Perseus 1.6.15.0 (https://maxquant.org/perseus/). Abundance was normalized against β-actin (Tb927.9.8880).

### Structure-Based Remote Homolog Detection for Tb927.8.6290

An AlphaFold model of the protein encoded by Tb927.8.6290 was downloaded from the Wheeler lab TriTryp AlphaFold database.^54^ This model was queried against the Protein Data Bank^55^ (PDB) with the PDBeFold webserver,^30,31^ with the query/target lowest acceptable match reduced set at 50% (Figure S6). The model was also screened against the PDB and AlphaFold Protein Structure Database^34^ with the DALI protein structure comparison server^32,33^ (Figures S6 and S7). Human inositol-tetrakisphosphate 1-kinase (ITPK1) was the top-ranked protein in all searches. Our analysis was based on the highest matching PDB structure identified from searches of PDBeFold. The AlphaFold model of Tb927.1.3300 was also queried against the PDB with PDBeFold, as described above, with the MTMR2 structure as the top-ranked hit (PDB ID: 1m7r).^39^

The matched structure of IPTK1 PDB ID: 2qb5 (chain B)^37^ was superimposed onto the Tb927.8.6290 model using the PDBeFold structure-based alignment with Jalview^56^ and UCSF Chimera.^57^ Superpositions of the individual domains of ITPK1 against *Tb*ITPK1 were generated with the UCSF Chimera^57^ Match Maker tool by restricting the target residues to the relevant domains.

### Docking and Resistance Mutant Modeling

Resistance mutations were mapped to the Tb927.8.6290 model with Jalview^56^ and UCSF Chimera^57^ to provide an indication of the likely binding site of the compounds. Compounds **38** and **69** were then blind-docked into the TriTryp AlphaFold model of Tb927.8.6290 with SwissDock^58,59^ in “Accurate” mode. The candidate poses were visualized in UCSF Chimera^57^ and we observed that the top-ranked pose clusters for both compounds were located in the ATP binding site and were proximal to the sites of the resistance mutations. The consistency between these independent features suggests that the docking algorithm has determined an accurate pose, and so we selected the top-ranked pose for each compound to proceed with modeling of the resistance mutations.

Models of the resistance mutants were generated from the apo model of Tb927.8.6290 with the UCSF Chimera^57^ swapaa tool and the built-in Dunbrack rotamer libraries.^60^ The swapaa tool can introduce only one mutation at a time, and so the double mutant was constructed serially. We found that the optimal rotamer for F241 in the V241F/A258V double mutant was influenced by the A258V substitution—but not vice versa—and so the model produced by introducing A258V before V241F was our preferred model. Compounds **38** and **69** were overlaid on the resistance mutant models in their wild-type poses and clashes were identified with UCSF Chimera findclash. In addition to clash detection, since proline mutations can have a profound effect on protein dynamics, we assessed the effect of the A258P resistance mutation with the DynaMut algorithm.^38^

## Supporting Information Available

Additional chemistry experimental details; results related to compound physicochemical properties, compound cidality, mechanism of action studies, docking studies, and HPLC chromatograms of key compounds; and compound physicochemical properties and mechanism-of-action studies (PDF)
Extended data sets for genome-wide genetic screens and whole-genome sequencing (XLSX)
Docking studies with compounds **38** and **69** and STRINGS for all compounds (CSV)
Molecular docking of ADP (PDB)
Molecular docking of compound **38** (PDB)
Molecular docking of compound **69** (PDB)
Molecular docking of the TbITPK1 A258P mutant (PDB)
Molecular docking of the TbITPK1 model (PDB)
Molecular docking of TbITPK1 V241F A258V (PDB)

## Supplementary Material

jm3c00509_si_003.csv

jm3c00509_si_004.pdb

jm3c00509_si_005.pdb

jm3c00509_si_006.pdb

jm3c00509_si_007.pdb

jm3c00509_si_008.pdb

jm3c00509_si_009.pdb

## Notes

Ethics: all regulated procedures on living animals were carried out under the authority of a project license issued by the Home Office under the Animals (Scientific Procedures) Act 1986, as amended in 2012 (and in compliance with EU Directive EU/2010/63). License applications will have been approved by the University’s Ethical Review Committee (ERC) before submission to the Home Office. The ERC has a general remit to develop and oversee policy on all aspects of the use of animals on university premises and is a subcommittee of the University Court, its highest governing body.

## Notes

Sequencing data has been submitted to the European Nucleotide Archive under the project number PRJEB55780.

## Abbreviations Used

CL_int_: intrinsic clearance
CNS: central nervous system
HAT: human African trypanosomiasis
HTS: high-throughput screening
ITPK1: inositol-tetrakisphosphate 1-kinase
MW: molecular weight
PI: phosphatidylinositol
RNAi: RNA interference
SAR: structure–activity relationship
SI: selectivity index
TPP: target product profile
TPSA: topological polar surface area
*Tb*: *Trypanosoma brucei*
*Tb*GSK3: *Trypanosoma brucei* glycogen synthase kinase 3.
