# Supplementary material for: Development of a 2,4-Diaminothiazole Series for the Treatment of Human African Trypanosomiasis Highlights the Importance of Static–Cidal Screening of Analogues
Source: J Med Chem. 2023 Jun 21;66(13):8896–916. doi: 10.1021/acs.jmedchem.3c00509 (PMC10350920; doi:10.1021/acs.jmedchem.3c00509)
Supplement: Supplementary file 1 — jm3c00509_si_001.pdf [file jm3c00509_si_001.pdf]

Supporting Information

Development of a 2,4-diaminothiazole series for the treatment of human African trypanosomiasis highlights the importance of static-cidal screening of analogues

Laura A. T. Cleghorn[1,*] Richard J. Wall[2,*], Sébastien Albrecht[1], Stuart A. MacGowan[3] , Suzanne Norval[1], Manu De Rycker[1], Andrew Woodland[1], Daniel Spinks[1], Stephen Thompson[1], Stephen Patterson[2], Victoriano Corpas Lopez[2], Gourav Dey[2], Iain T. Collie[1], Irene Hallyburton[1], Robert Kime[1], Frederick R. C. Simeons[1], Laste Stojanovski[1], Julie A. Frearson[1], Paul G. Wyatt[1], Kevin D. Read[1], Ian H. Gilbert[1], Susan Wyllie[2].

[1]Drug Discovery Unit, Wellcome Centre for Anti-infectives Research, Division of Biological Chemistry and Drug Discovery, University of Dundee, Dow Street, Dundee, DD1 5EH, UK

[2]Wellcome Centre for Anti-infectives Research, Division of Biological Chemistry and Drug Discovery, School of Life Sciences, University of Dundee, Dow Street, Dundee, DD1 5EH, UK

[3]Division of Computational Biology, School of Life Sciences, University of Dundee, Dow Street, Dundee, DD1 5EH, UK

Chemical synthesis

General methods Chemicals and solvents were purchased from Aldrich Chemical Co., Alfa Aesar, Fluorochem, Apollo and Fisher Chemicals and were used as received, unless otherwise stated. Air- and moisture-sensitive reactions were carried out under an inert atmosphere of argon in oven-dried glassware. Analytical thin-layer chromatography (TLC) was performed on precoated TLC plates (layer 0.20 mm silica gel 60 with fluorescent indicator UV254, from Merck). Developed plates were air-dried and analysed under a UV lamp (UV 254/365 nm) and/or $KMnO_4$ was used for visualization. Flash column chromatography was performed on an automated purification system (Teledyne ISCO Combiflash Companion or Combiflash Retrieve) using Grace Resolve prepacked silica gel cartridges (230-400 mesh, 40-63 µm, varying sizes depending on reaction scale). ^{1}H and ^{13}C NMR spectra were recorded on a Bruker Advance II 500 spectrometer operating at 500.1 and 125.8 MHz (unless otherwise stated) using $CDCl_3$ or DMSO-d_6 solutions. Chemical shifts (δ) are expressed in ppm recorded using the residual solvent as the internal reference in all cases. Signal splitting patterns are described as singlet (s), doublet (d), triplet (t), multiplet (m), broadened (b) or a combination thereof. Coupling constants (*J*) are quoted to the nearest 0.1 Hertz (Hz). LC-MS analyses were performed with either an Agilent HPLC 1100 series connected to a Bruker Daltonics MicroTOF or and Agilent Technologies 1200 series HPLC connected to an Agilent Technologies 6130 quadrupole spectrometer, where both instruments were connected to an agilent diode array detector. LC-MS chromatographic separations were conducted with a Water Xbridge C18 column, 50 mm x 2.1 mm, 3.5 µm particle size; using either methanol, methanol/water (95:5) or water /acetonitrile (1:1) + 0.1% formic acid as the mobile phase; linear gradient from 80:20 to 5:95 over 3.5 min and then held for 1.5 min; flow rate of 0.5 $mLmin^{-1}$. The purity of all synthesised compounds was confirmed to be >95% using this LC-MS method (TIC and UV) and this analytical LC-MS system. High resolution electrospray measurements were performed on a Bruker MicroTof mass spectrometer. Microwave-assisted chemistry was performed using a Biotage initiator microwave synthesizer.

General procedure A: Three-component cyclisation reaction

2-Benzylisothiouronium bromide (1 mol eq.), DIPEA (1.1 mol eq.), DMF (1.5 $mLmmol^{-1}$) and cyclohexylisothiocyanate (1.05 mol eq.) were stirred at rt for 6 h. Following this α-bromoketone (1.2 mol eq.) and DIPEA (0.35 mL, 2 mol eq.) were added, the reaction stirred at rt for a further 16 h then quenched with a 10% (v/v) HCl solution and partitioned into EtOAc, the organic fractions were dried over $MgSO_4$, filtered and the solvent removed in-vacuo. The crude mixture was purified by column chromatography eluting with petroleum ether (40 - 60°C): EtOAc, 8:2, to afford the desired product.

1-(4-Amino-2-(cyclohexylamino)thiazol-5-yl)-2-bromo-2-methylpropan-1-one

Cyclohexyl isothiocyanate (0.07 mL, 0.55 mmol) and 1,3-dibromo-3-methylbutan-2-one (120 mg, 0.5 mmol) were reacted as described in general procedure A to afford the title compound, 126 mg, 73%; δ_H (300 MHz, $CDCl_3$) 5.48 (d, *J* = 7.3 Hz, 1H, NH), 3.43 (sep, *J* = 4.6 Hz, 1H, CH), 2.15-2.12 (m, 1H, CH_2), 2.00 (s, 6H, 2 x CH_3), 1.83-1.77 (m, 2H, CH_2), 1.70-1.66 (m, 1H, CH_2), 1.48-1.46 (m, 1H, CH_2), 1.44-1.42 (m, 1H, CH_2), 0.93-0.88 (m, 3H, CH_2); LCMS (ES+): m/z (%) 346 and 348, ^{35}Cl and ^{37}Cl $[M+H]^+$ t_R : 4.1-4.2 (20-90% MeCN, basic).

***N'*-(Cyclohexylcarbamothionyl)-*N*,*N*-dimethylformimidamide**

1-Cyclohexyl thiourea (1.58 g, 10 mmol) and *N*,*N*-dimethylacetate (1.59 mL, 12 mmol) were heated in RB flask for 5 h at 100 °C, the mixture cooled to rt and excess solvent removed *in vacuo*. Upon standing a pale orange crystalline solid formed, 1.5 g, 70% yield (mixture of E and Z isomers, 9:4 by ^1H NMR, 500 MHz, $CDCl_3$) that was used without further purification or separation of isomers. LCMS (ES+): m/z (%) 314 $[M+H]^+$ t_R : 318 (20-95% MeCN, acidic).

1-Bromo-4-hydroxy-4-methylpentan-2-one

Bromine (0.092 mL, 1.8 mmol) was added to a solution of hydroxypentanone (0.13 mL, 2 mmol) in methanol (anhydrous, 2 mL) at -5 °C. The solution was stirred for 1 h, then warmed to rt over 30 min, poured over ether and washed with H_2O (3 x 20 mL), dried over $MgSO_4$,

filtered and the solvent removed *in vacuo* to afford a colourless oil (0.34 g, 100%) which was used without further purification due to volatility.

General procedure B: Three-component cyclisation to form bromoethanone intermediate

1-(4-Amino-2-((3,4-difluorophenyl)amino)thiazol-5-yl)-2-bromoethanone

Prepared following general procedure A, 352 mg, 67%; δ_H (500 MHz, MeOD) 7.93 (ddd, J = 13.1, 7.2 and 2.5 Hz, 1H, ArH), 7.28-7.26 (m, 2H, ArH), 4.20 (s, 1H, CH_2), 4.00 (s, 1H, CH_2); LCMS (ES+): m/z (%) 348 and 350 ^{35}Cl and ^{37} Cl $[M+H]^+$ t_R : 4.6 (5-95% MeCN, basic).

General procedure C: Conversion of bromoethanones into ether ethanones

Sodium hydride (7 mol eq.) was added portion wise to a solution of alcohol (3 mol. eq.) in THF (1 mL/mmol) at rt and the reaction stirred for 30 min. 1-(4-amino-2-((3,4-difluorophenyl)amino)thiazol-5-yl)-2-bromoethanone (1 mol eq.) was added and the mixture refluxed for 1 h. Excess solvent was removed *in vacuo* and the crude residue partitioned between DCM/H_2O and passed through a hydrophobic frit and the organic solvent removed in vacuo. Column chromatography eluting with petroleum ether (40 – 60 °C)/EtOAc 1:1 afforded the desired product.

2-Bromo-*N*-methoxy-*N*-methylacetamide

Bromoacetylbromide (2 mL, 23 mmol) was added drop-wise to a stirred solution of *N*,*O*-dimethylhydroxylamine.HCl (2.24 g, 23 mmol), K_2CO_3 in DCM (anhydrous, 10 mL) cooled to 0°C. The mixture was stirred at 0 °C for 1 h and rt for 30 min, stopped, quenched with brine and passed through a hydrophobic frit, the organic solvent removed *in vacuo* to afford the title compound as a colourless oil which was used without further purification. δ_H (500 MHz, $CDCl_3$) 4.03 (s, 2H, CH_2), 3.81 (s, 3H, OCH_3), 3.26 (s, 3H, CH_3).

***N*-Methoxy-*N*-methyl-2-((1,1,1-trifluoropropan-2-yl)oxy)acetamide**

Sodium hydride (2.0 g, 60% wt mineral oil) was added portion wise to a solution of 1,1,1 trifluoropropan-2-ol (4.7 g, 41 mmol) in Et_2O (anhydrous, 20 mL) at rt. The solution cooled to 0 °C and 2-bromo-*N*-methoxy-*N*-methylacetamide in Et_2O (10 mL, anhydrous) added slowly over 15 min. The reaction was stirred at 0 °C for 30 min and quenched with H_2O, partitioned between Et_2O and H_2O and the organic layer dried over $MgSO_4$ and excess solvent removed *in vacuo*. Column chromatography eluting with diethyl ether afforded the title compound as a colourless oil which was used without further purification. δ_H (500 MHz, $CDCl_3$) 4.56 (d, J = 16.1 Hz, 1H, CH_2), 4.44 (d, J = 16.1 Hz, 1H, CH_2), 3.99 (sep, J = 6.5 Hz, 1H, CH), 3.71 (s, 3H, CH_3), 3.22 (s, 3H, CH_3), 1.47 (d, J = 6.5 Hz, 3H, CH_3); LCMS (ES+): m/z (%) 216 $[M+H]^+$ t_R : 4.2-4.3 (5-95% MeCN, basic).

1-Chloro-3-((1,1,1-trifluoropropan-2-yl)oxy)propan-2-one

Methyl lithium (3.05 mL, 1.6 M in Et_2O) was added over a period of 15 min to a solution of chloroiodomethane (0.51 mL, 9 mmol) and *N*-methoxy-*N*-methyl-2-((1,1,1-trifluoropropan-2-yl)oxy)acetamide (1.0 g, 7.0 mmol) in Et_2O and cooled to -78 °C. The reaction stirred for 30 min and quenched with a sat. solution of ammonium chloride. Extraction into Et_2O afforded the title compound, 1.0 g, 82%; δ_H (500 MHz, $CDCl_3$) 4.52 (d, J = 17.0 Hz, 1H, CH_2), 4.44 (d, J = 17.0 Hz, 1H, CH_2), 4.32 (d, J = 16.1 Hz, 1H, CH_2), 4.26 (d, J = 16.1 Hz, 1H, CH_2), 3.87 (sep, J = 6.5 Hz, 1H, CH), 1.45 (dd, J = 6.5 and 0.7 Hz, 3H, CH_3); LCMS (ES+): m/z (%) 244 and 246 ^{35}Cl and $^{37}Cl[M+MeCN]^+$ t_R : 4.7-4.8 (5-95% MeCN, basic);

(4-Amino-2-((4-bromophenyl)amino)thiazol-5-yl)(2,6-difluorophenyl)methanone

Prepared following general procedure A, beige solid, 1.85g, 90%; δ_H (300 MHz, d_6-DMSO) 10.96 (s, 1H, NH), 8.22 (bs, 2H, NH_2), 7.49-7.63 (m, 5H, ArH), 7.21 (m, 2H, ArH); LCMS (ES+): m/z (%) 410/412 $[M+H]^+$ t_R : 5.10 (5-95% MeCN, basic).

General procedure D: Three-component cyclisation reaction

3,5-Dimethyl-1*H*-pyrazole-1-carboximidamide nitrate (1 mol eq.), DIPEA (1.1 mol eq.), DMF (1.5 $mLmmol^{-1}$) and cyclohexylisothiocyanate (1.05 mol eq.) were stirred at rt for 6 h. Following this 1-chloro-3-((1,1,1-trifluoropropan-2-yl)oxy)propan-2-one (1.2 mol eq.) and DIPEA (0.35 mL, 2 mol eq.) were added, the reaction stirred at rt for a further 16 h then quenched with a 10% (v/v) HCl solution and partitioned into EtOAc. The organic fractions were dried over $MgSO_4$, filtered and the solvent removed *in vacuo*. The crude mixture was purified by column chromatography eluting with petroleum ether (40 – 60 °C): EtOAc (8:2) to afford the desired product.

General procedure E: displacement of bromoethanone with amines

DIPEA (1.15 eq) then a solution of the required isothiocyanate (0.22 mmol) in DMF (2 mL) was added successively to a solution of thiouronium salt (0.22 mmol) in DMF (2 mL), the reaction mixture was stirred at rt for 16 h, then α α'-dibromoketone (0.35 mmol, 1.6 eq) in DMF (1 mL) was added and the reaction stirred for a further 1.5 h, treated with the desired amine (0.44 mmol, 2 eq.) and stirred for a further 2 h or microwave heated at 100 °C for 30 min in presence of Na_2CO_3 (3 eq). The reaction was quenched with aq. $NaHCO_3$, extracted into EtOAc and washed with brine (2x). The organics were separated, dried over Na_2SO_4 and excess solvent removed *in vacuo*. Column chromatography eluting with 0 to 15% MeOH in CH_2Cl_2 afforded the desired compound.

Scheme 1

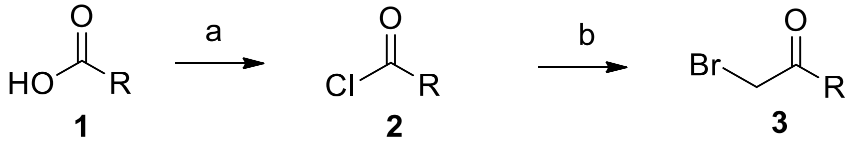

(a) SO_2Cl, 50 °C, 2 h (b) (i) TMS-diazomethane, MeCN, 0 °C to rt, 1 h (ii) HBr, 0 °C, 10 min.

Scheme 2

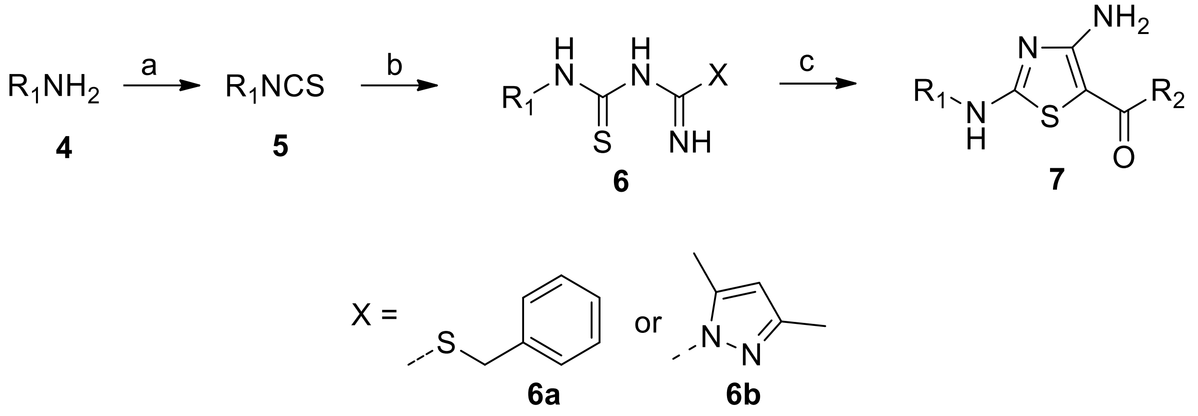

(a) DMF, 0°C to rt (b) benzyl carbaminidothioate hydrobromide salt **6a** or 3,5-dimethyl-1*H*-pyrazole-1-carboximidamide nitrate salt **6b**, DIPEA, DMF, rt, 2-4 h (c) α-bromoketones, DIPEA, rt, 2h.

Scheme 3

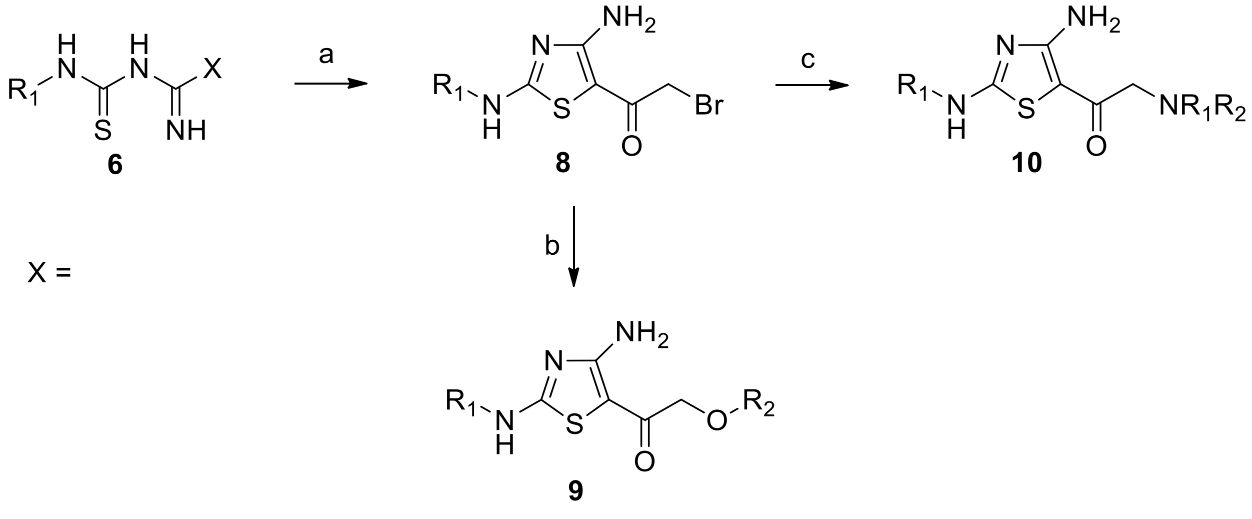

(a) 1,3-dibromopropan-2-one, DIPEA, DMF, rt, 2 h (b) NaH, Et_2O, 0°C to rt (c) NHR_1R_2 (2eq.), rt, 2 h or µW, 100 °C, 30 min, Na_2CO_3 (3 eq.).

Scheme 4

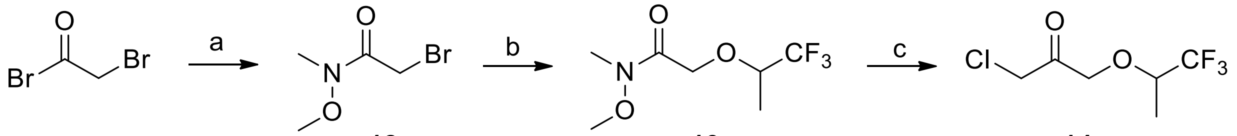

(a) *N*,*O*-dimethylhydroxylamine hydrochloride, 0°C, 1 h to rt, 30 min (b) NaH, Et_2O, 1,1,1-trifluoropropan-2-ol, 0 °C to rt (c) Chloroiodomethane, MeLi (1.6 M in Et_2O), -78 °C, 1h.

Supplementary Figures

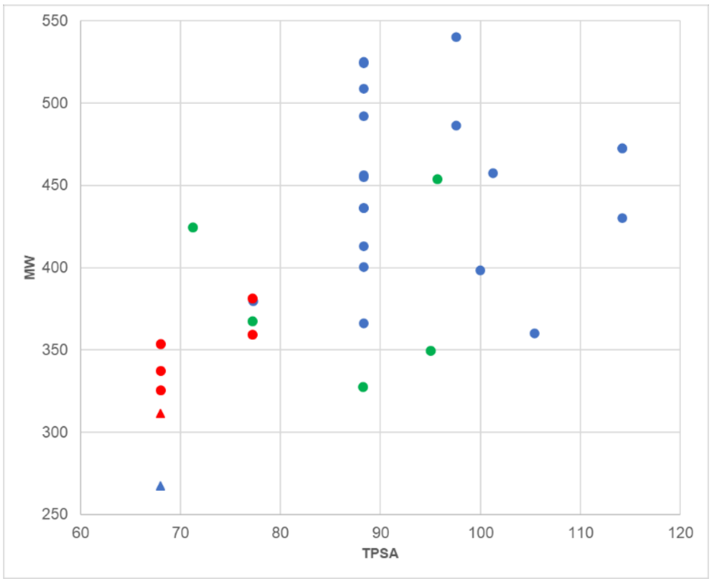

Figure S1: Molecular weight (MW) plotted against TPSA (topological polar surface area for compounds profiled for mouse brain penetration. Also see also Table **S2**. Compounds were classified as having a B:B of <1 (blue), 1-3 (green) and >3 (red). All highly penetrant compounds had a TPSA of <80 and MW <400, within the generally accepted rules for CNS penetration. Compound **18**, which had a lower B:B than expected and the structurally-related **38** are shown as red and blue triangles, respectively.

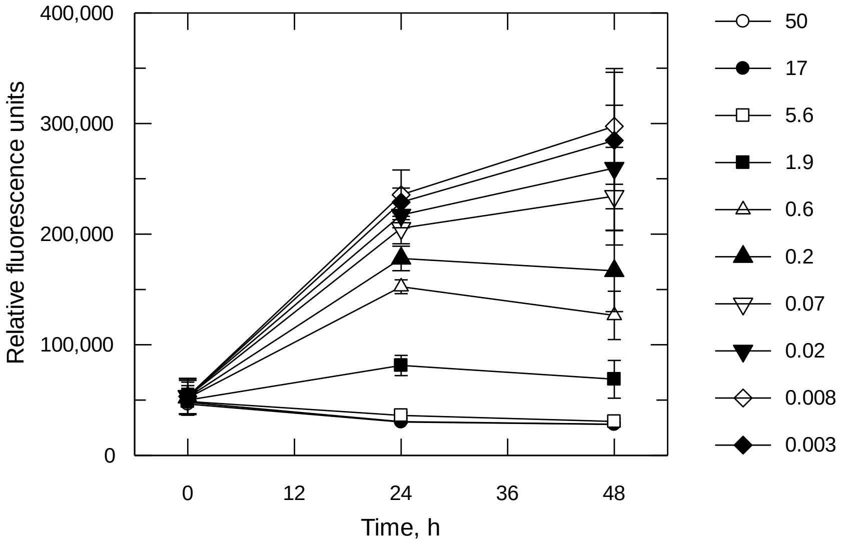

Figure S2: Static-cidal assay with compound **16**. Concentrations of compound indicated in µM.

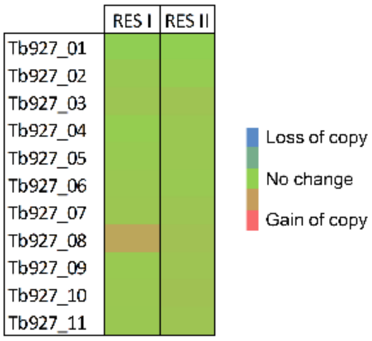

Figure S3: Copy number variations in compound 38-resistant cell lines.

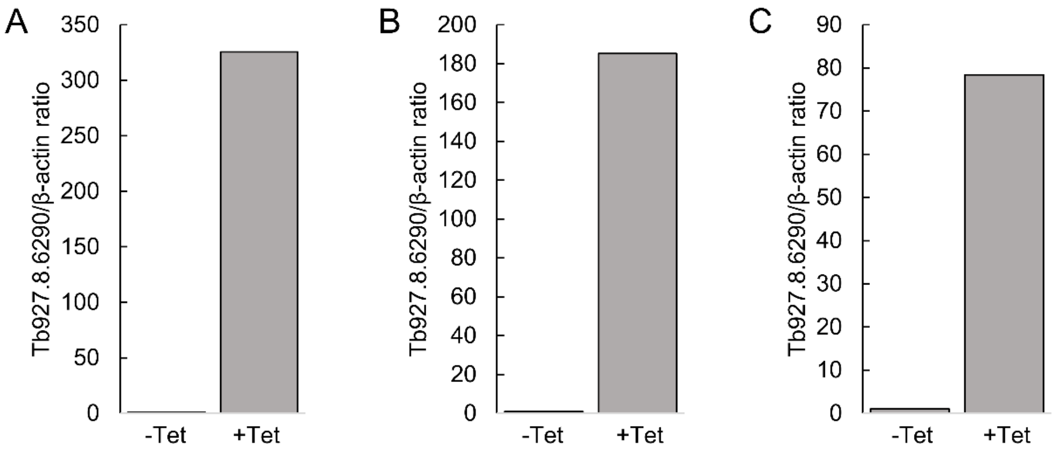

Figure S4: Label-free quantitative proteomics confirm overexpression of the hypothetical protein encoded by Tb927.8.6290 in transgenic trypanosomes. Relative levels of overexpression of wild-type Tb927.8.6290 (A), Tb927.8.6290 bearing an A258P mutation (B) and Tb927.8.6290 bearing V241F and A258V double mutation (C). Normalised to β-actin.

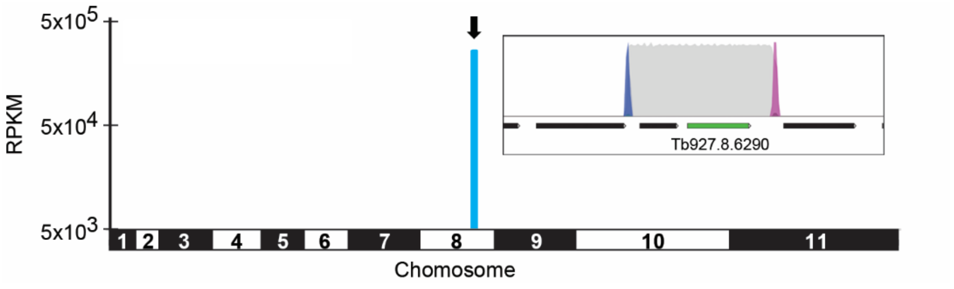

Figure S5: Genome-wide overexpression library screen with compound 69. Compound **69** screened against our genome-wide overexpression library at a concentration equivalent to 2x the established EC_{50} (300 nM). Genome-wide map showing the main hits are shown. RPKM: reads per kilobase of transcript per million mapped reads. Insert focuses on the top fragment hit of the overexpression library screen containing two full coding sequences including Tb927.8.6290. Gene of interest highlighted in green, other protein-coding regions in black. Blue and pink peaks are OE construct forward and reverse barcodes (in the sense orientation), respectively. Grey peaks are all other reads. See also Table **S6**.

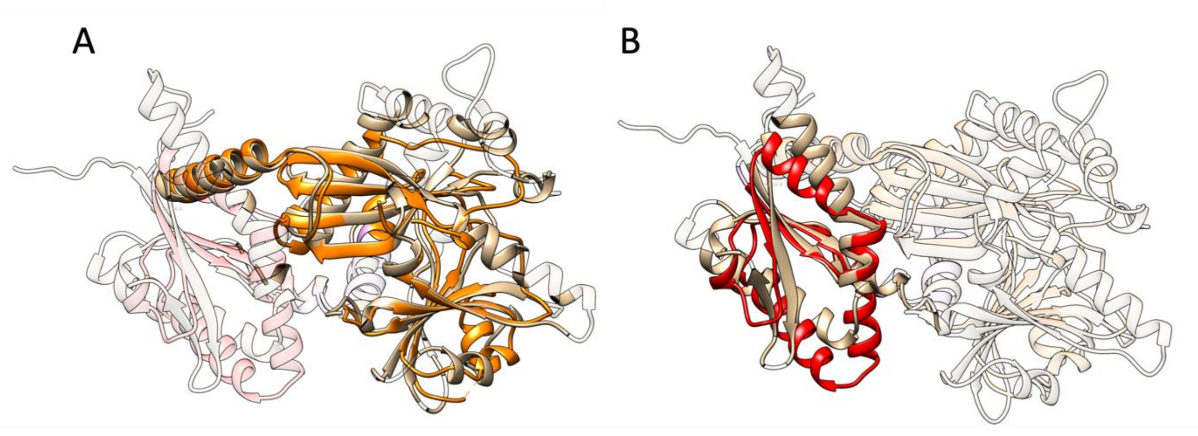

Figure S6: Structural alignment of Tb927.8.6290: TriTrypAF model of Tb927.8.6290 (tan) with individual domains of human inositol-tetrakisphosphate 1-kinase (ITPK1; PDB ID: 2qb5 chain B [1]. A) Alignment of the ATP-grasp domain (orange). B) Alignment of the ITPK1 N-term domain (red). Alignments were generated using UCSF Chimera MatchMaker tool [2]. Regions that are not structurally aligned regions are rendered transparent.

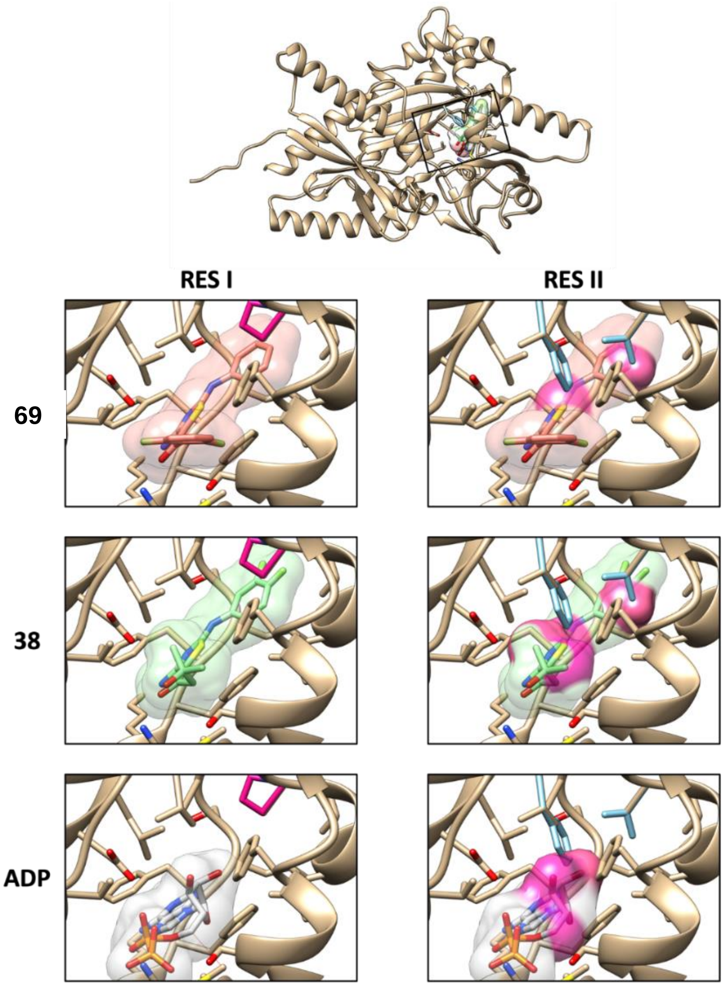

Figure S7: Effect of RES I and II resistance mutations on binding of 38, 69 and ADP. Mutants were modelled using the UCSF Chimera swapaa tool starting from the TriTrypAF model of Tb927.8.6290 (see Experimental Section for details). The original AlphaFold model of the wild-type is shown in tan and RES I and II mutants are coloured pink and light blue, respectively. Compounds **38** and **69** were docked into the RES I/II models using the docking poses predicted for the wild-type without optimisation. ADP positioning was achieved by superimposing the *Tb*ITPK1 TriTrypAF model and human ITPK1 PDB ID: 2qb5 (chain B) without optimisation. Magenta colouring of the ligand molecular surfaces indicates regions that are clashing with the resistance mutant models, except in the RES I:ADP complex were a solitary clash – arising from a wild-type residue and likely an artefact of the placement routine – is indicated by a red bond.

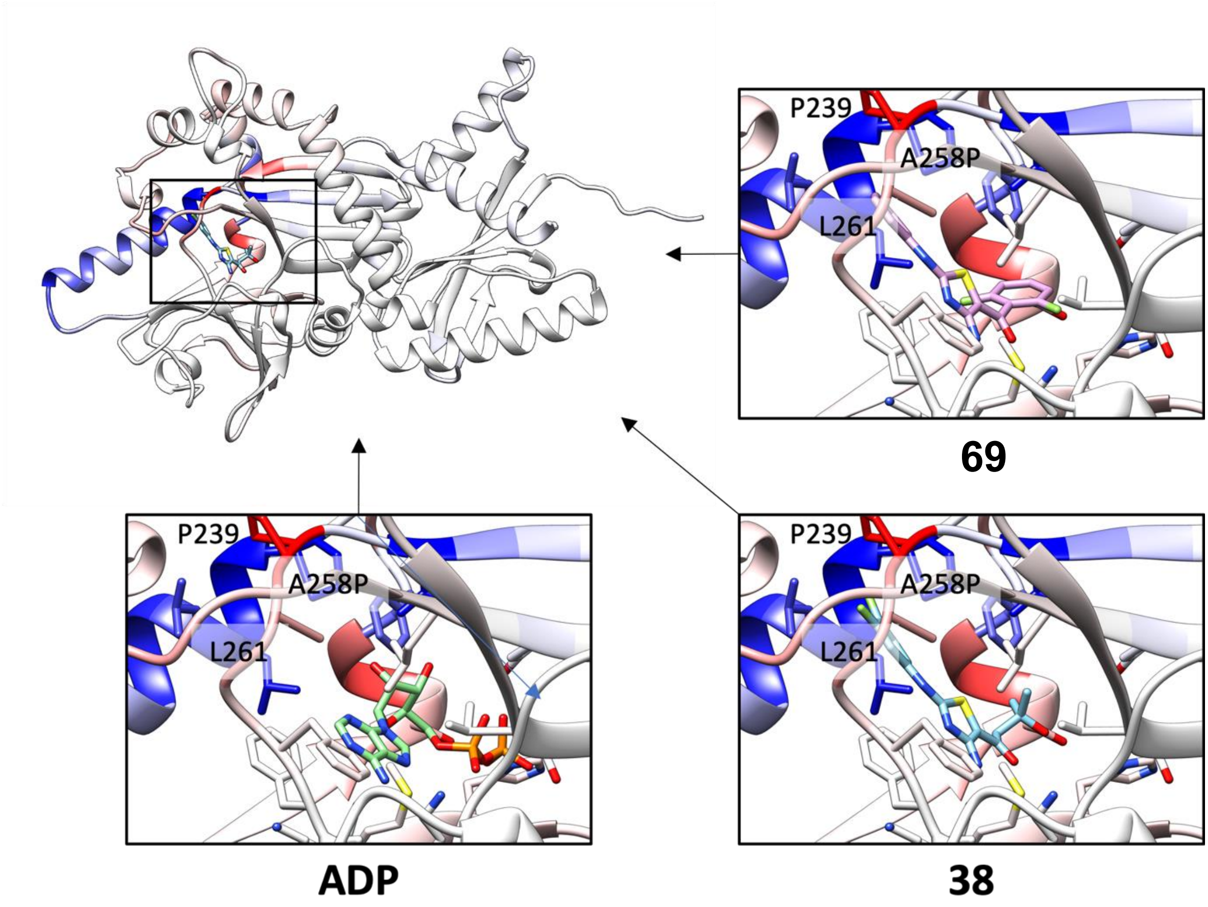

Figure S8: The impact of the RES I mutation A258P on protein dynamics and its effect on ligand binding. The protein is coloured by the predicted change in vibrational entropy of each residue ($\Delta\Delta S_{vib}$) from DynaMut [3] where blue indicates regions of decreased flexibility ($\Delta\Delta S_{vib}$ <0) and red regions of increased flexibility ($\Delta\Delta S_{vib}$ >0). The aromatic R_1 group of compounds **38** and **69** interacts with residues on the A258P rigidified helix, which could inhibit binding by disfavouring local rearrangement to accommodate the ligands. In contrast, ADP is not bound to this area of increased rigidity and so may ADP/ATP binding may be less sensitive to this mutation. Figure created using UCSF Chimera [2].

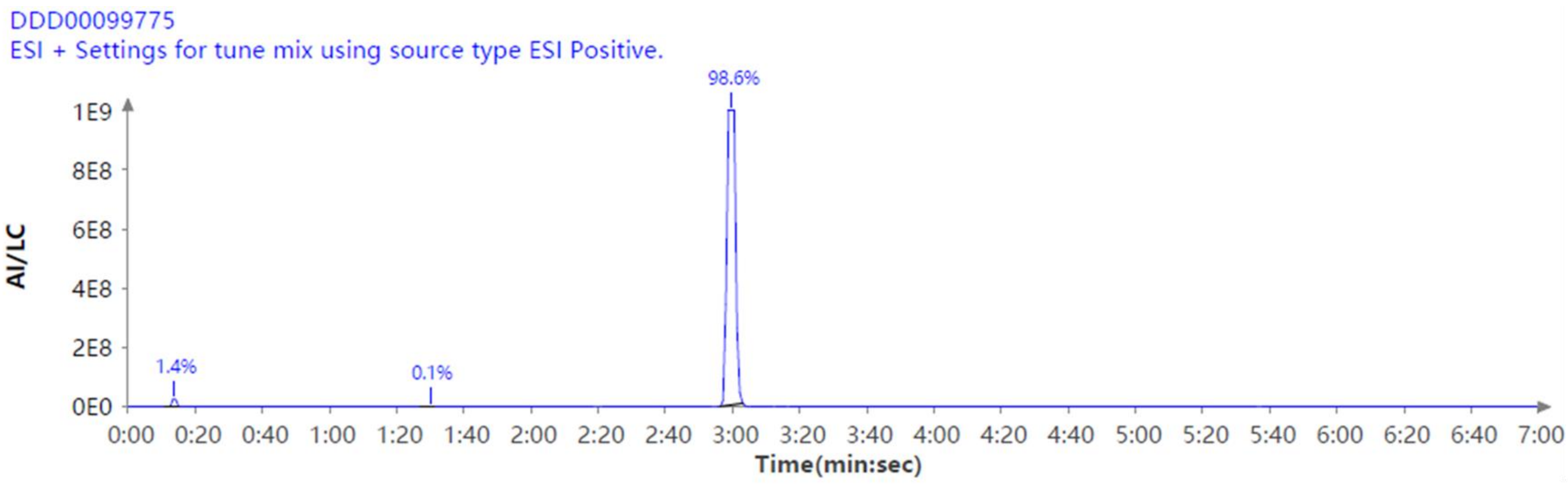

Time (Peak Maximum M:S/Minute-s)	Maximum AI/LC ( )	Time (Peak Centroid M:S/Minute-s)	Peak Area	% Peak Area	Peak Resolution
0:13	2.6E7	0:14	4.2E7	1.4	1.7
1:30	1.5E6	1:30	2.1E6	0.1	1.5
2:59	9.9E8	2:60	3E9	98.6	2.9

Figure S9: HPLC chromatogram of compound 16.

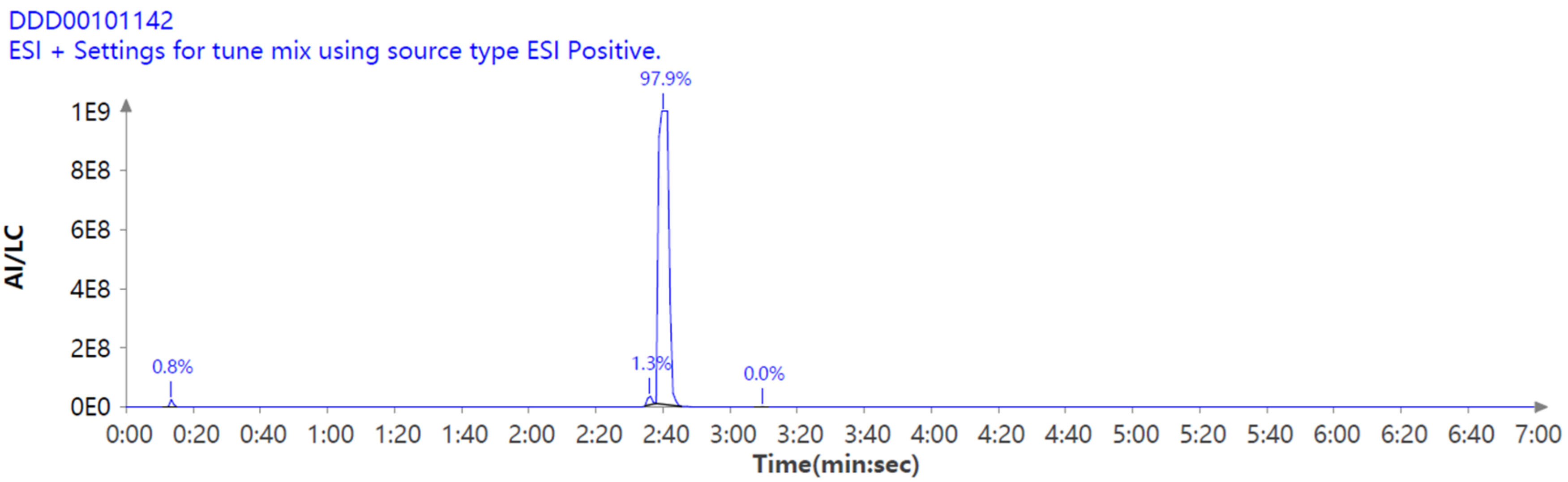

Time (Peak Maximum M:S/Minute-s)	Maximum AI/LC ( )	Time (Peak Centroid M:S/Minute-s)	Peak Area	% Peak Area	Peak Resolution
0:13	2.5E7	0:13	2.9E7	0.8	1.0
2:36	2.8E7	2:36	4.8E7	1.3	2.5
2:40	9.9E8	2:40	3.6E9	97.9	3.5
3:10	5.1E5	3:10	4.2E5	0.0	0.8

Figure S10: HPLC chromatogram of compound 38.

Supplementary Tables

Table S1: clogP and PSA values for selected analogues.

Compound	clogP	PSA	Compound	clogP	PSA
**17**	2.5	77	**43**	3.0	77
**18**	2.7	68	**44**	2.4	77
**19**	2.6	68	**45**	3.0	77
**20**	3.5	68	**46**	2.8	77
**21**	4.4	68	**47**	3.5	77
**16**	3.4	68	**48**	2.7	77
**22**	3.4	68	**49**	3.6	77
**23**	4.2	68	**50**	3.3	77
**24**	3.7	68	**51**	3.5	77
**25**	**2.3**	81	**52**	3.8	77
**26**	3.7	77	**53**	2.9	96
**27**	3.4	68	**54**	3.2	86
**28**	4.2	68	**55**	2.2	95
**29**	3.4	68	**56**	3.0	90
**30**	3.4	68	**57**	2.3	99
**31**	3.4	68	**58**	2.2	71
**32**	3.9	42	**59**	2.7	71
**33**	3.9	42	**60**	3.3	71
**34**	2.6	68	**61**	2.7	71
**35**	2.8	68	**62**	1.8	80
**36**	3.0	68	**63**	2.6	80
**37**	3.1	68	**64**	2.0	80
**38**	2.7	88	**65**	2.1	80

**39**	2.8	77	**66**	2.4	80
**40**	3.5	68	**67**	2.7	71
**41**	3.0	68	**68**	2.7	71
**42**	2.8	77			

Table S2: Physicochemical properties of compounds with measured mouse Brain: Blood ratios. Key compounds highlighted in red.

Compound	B:B	MW	logP	TPSA
70	0.02	430.49	2.3	114
71	0.03	486.54	2.9	98
72	0.04	472.57	3.3	114
73	0.04	472.57	3.3	114
74	0.05	413.32	2.3	88
75	0.05	455.4	3.5	88
18	0.06	267.39	2.7	68
76	0.06	525.4	3.9	88
77	0.07	436.52	3.0	88
78	0.09	508.94	3.5	88
79	0.1	524.51	3.9	88
80	0.1	540.51	3.7	97
81	0.1	492.49	3.1	88
82	0.2	360.5	1.6	105
83	0.2	456.51	2.9	88
84	0.3	457.5	2.1	101
85	0.3	436.52	2.9	88
86	0.4	436.52	2.9	88
87	0.5	400.54	2.5	88
88	0.8	398.53	3.1	100
89	0.9	366.52	2.3	88
51	0.9	379.79	3.5	77
90	1	453.86	3.4	96
91	1.2	349.33	2.2	95
92	1.4	424.37	3.2	71
38	1.4	327.35	2.7	88
93	1.6	367.41	3.2	77
16	3.3	337.39	3.4	68
94	3.4	381.32	3.3	77
95	3.9	353.48	3.6	68
37	4.7	311.35	3.1	68
96	4.8	359.32	2.5	77
97	5.2	325.38	3.0	68

Brain: Blood ratio <1
Brain: Blood ratio 1-3
Brain: Blood ratio >3

Table S3: Extended hit list for compound 38 RIT-seq analysis (>1000 RPKM 'barcoded reads' cut-off).

Table S4: Extended hit list for compound 69 RIT-seq analysis (>1000 RPKM 'barcoded reads' cut-off).

Table S5: Fragment hits from screening the *T. brucei* overexpression library with compound 38. Fragments containing genes with >500 RPKM are shown.

Table S6: Summary of mutations found in compound 38-resistant lines.

Table S7: Fragment hits from screening the *T. brucei* overexpression library with compound 69. Fragments containing genes with >500 RPKM are shown.

References

1. Chamberlain, P.P., et al., *Integration of inositol phosphate signaling pathways via human ITPK1*. J Biol Chem, 2007. **282**(38): p. 28117-25.
2. Pettersen, E.F., et al., *UCSF Chimera--a visualization system for exploratory research and analysis*. J Comput Chem, 2004. **25**(13): p. 1605-12.
3. Rodrigues, C.H., D.E. Pires, and D.B. Ascher, *DynaMut: predicting the impact of mutations on protein conformation, flexibility and stability*. Nucleic Acids Res, 2018. **46**(W1): p. W350-W355.
4. T. T. Wager, R.Y.C., T. W. Butler, *Morpholine derivatives as D3 dopamine antagonists and their preparation, pharmaceutical compositions and use in the treatment of diseases*. 2008.
5. Laha, J.K., et al., *Structure-activity relationship study of 2,4-diaminothiazoles as Cdk5/p25 kinase inhibitors*. Bioorg Med Chem Lett, 2011. **21**(7): p. 2098-101.
